# CD28 Signaling Drives Notch Ligand Expression on CD4 T Cells

**DOI:** 10.3389/fimmu.2020.00735

**Published:** 2020-05-07

**Authors:** Ankita Mitra, Sudarvili Shanthalingam, Heather L. Sherman, Khushboo Singh, Mine Canakci, Joe A. Torres, Rebecca Lawlor, Yong Ran, Todd E. Golde, Lucio Miele, Sankaran Thayumanavan, Lisa M. Minter, Barbara A. Osborne

**Affiliations:** ^1^Department of Veterinary and Animal Sciences, University of Massachusetts Amherst, Amherst, MA, United States; ^2^Department of Chemistry, University of Massachusetts Amherst, Amherst, MA, United States; ^3^Center for Translational Research in Neurodegenerative Disease, University of Florida, Gainesville, FL, United States; ^4^School of Medicine, Department of Genetics, LSU Health Sciences Center, New Orleans, LA, United States

**Keywords:** Notch1, DLL1, DLL4, JAG1, CD28, *cis*

## Abstract

Notch signaling provides an important cue in the mammalian developmental process. It is a key player in T cell development and function. Notch ligands such as Delta-like ligands (DLL) 1, 3, 4, and JAG1, 2 can impact Notch signaling positively or negatively, by *trans*-activation or *cis*-inhibition. *Trans* and *cis* interactions are receptor-ligand interaction on two adjacent cells and interaction on the same cell, respectively. The former sends an activation signal and the later, a signal for inhibition of Notch. However, earlier reports suggested that Notch is activated in the absence of Notch ligand-expressing APCs in a purified population of CD4 T cells. Thus, the role of ligands in Notch activation, in a purified population of CD4 T cells, remains obscure. In this study, we demonstrate that mature CD4 T cells are capable of expressing Notch ligands on their surface very early upon activation with soluble antibodies against CD3 and CD28. Moreover, signaling solely through CD28 induces Notch ligand expression and CD3 signaling inhibits ligand expression, in contrast to Notch which is induced by CD3 signaling. Additionally, by using decoys, mimicking the Notch extracellular domain, we demonstrated that DLL1, DLL4, and JAG1, expressed on the T cells, can *cis*-interact with the Notch receptor and inhibit activation of Notch. Thus, our data indicate a novel mechanism of the regulation of Notch ligand expression on CD4 T cells and its impact on activated Notch.

## Introduction

Notch signaling is one of the most conserved signaling pathways in eukaryotes and is critical throughout development in vertebrates as well as in invertebrates (1). Deregulation of Notch signaling is associated with a wide range of diseases including cancer (2). There are four different Notch receptors found in mammals, Notch1–4. Canonical Notch ligands fall into a category called Delta/Serrate/Lag-2 (DSL) ligands, named for their conserved DSL like domain. The ligands in mammals can be categorized into two groups, homologs of Drosophila Notch ligands Delta or Serrate. The Delta like ligands (DLL) in mammals are DLL1, DLL3, and DLL4 and Serrate like ligands are JAG1 and JAG2 (3, 4). In addition to containing a DSL domain, all five vertebrate ligands possess extracellular epidermal growth factor (EGF) like repeats. JAG1 and 2 also have a conserved cysteine rich domain. The mature Notch receptor also consists of an extracellular (ECD) with EGF like repeats, a transmembrane (TM) and an intracellular domain (ICD). The EGF like repeats in the extracellular domain of Notch receptors and ligands are primarily involved in the ligand-receptor interaction (3, 4). The number of EGF-like repeats in the ECD of mammalian Notch paralogs vary from 36 in Notch1 and 2 to 34 in Notch 3 and 29 in Notch4 (5).

Notch can be activated in T cells by interaction with canonical Notch ligands on antigen presenting cells (APC) such as dendritic cells and marginal zone B cells (6–9). However, in T cells, our lab and others have observed that activation of Notch is initiated by signaling through TCR and CD28 in the presumptive absence of ligands (10–15). In ligand dependent Notch signaling, binding of Notch ligand to the receptor is followed by two proteolytic cleavages. Prior to transit to the cell surface, Notch is cleaved in the *trans*-Golgi by furin protease. This cleavage, known as S1, results in the production of N-terminal ECD and a transmembrane peptide containing the TM and the ICD. In ligand dependent activation, mechanical stress by ligand-receptor interaction pulls the Notch ECD from the cell surface and exposes S2 site for cleavage (16, 17). Following ligand binding, the ECD separates from the TM subunit, unmasking an ADAM cleavage site (S2). This initiates cleavage by ADAM proteases which results in a conformational change that renders the transmembrane bound Notch peptide a substrate for the intramembranous γ-secretase complex. This final cleavage by γ-secretase, known as the S3 site, releases the transcriptionally active intracellular domain of Notch into cytosol (NICD). In canonical Notch signaling, this fragment translocates to the nucleus and associates with the DNA binding protein CSL resulting in Notch target gene transcription.

Earlier reports suggested that the two ADAM proteases, ADAM 10 and 17 are differentially involved in Notch processing (18). ADAM 10 is required for S2 cleavage in ligand dependent activation in mammals. In contrast, ADAM17 is suggested to cleave Notch in a ligand independent manner (18). However, it is still unclear how this preferential cleavage occurs. It is possible that unfolding of the ECD in the acidic environment of the endosomes unmasks the S2 site for ADAM17 cleavage in the context of ligand-independent activation (19).

In T cells, Notch plays a critical role in determining the cell fate at different stages of development, as well as function in the periphery. Interestingly, Notch ligands also play an important role in orchestrating these decisions. The lymphoid progenitors are steered toward the T cell lineage, avoiding a B cell fate, by the differential expression of Notch ligands (20, 21). In addition, DLL1-Notch interaction in thymus promotes survival of CD4- CD8- pre T-cells by regulation of cellular metabolism (22–24). Once released in the periphery, signaling through Notch regulates several functions of naïve mature T cells such as proliferation, and CD4 T cell polarization (11, 12, 25, 26). APCs can tailor responses of T cells toward a specific antigen by regulating Notch signaling through different Notch ligand interactions which, in turn, determines the choice between different T helper cell subsets (7). For example, the interaction of Notch with Delta like ligands have been associated with Th1 and Th17 polarization (27–29). Assisted by the cytokine milieu provided by the antigen presenting cells, another factor that can manipulate T cell responses toward specific pathogens is the strength of signaling through the TCR, determined by the amount of antigen presented on the major histocompatibility complex (MHC). TCR signal strength can regulate T helper cell polarization (30), and can control the differentiation of CD4 into effector and memory T cells (31). Th1 polarization is favored by a stronger TCR signal (29). Additionally, the extent of polarization is regulated by TCR signal strength determined by the dose of antigen (32). In *in-vitro* assays, this manipulation can result from the differential amount of antibodies engaging a component of the TCR complex (CD3) and the costimulatory molecule (CD28). Interestingly, increasing signal strength through CD3 leads to an increase in activated Notch and Notch, in turn, can also regulate the strength of TCR signal (11, 33). Although Winandy and Colleagues, recently published findings supporting ligand-independent activation of Notch in naïve CD4 T cells, the role, if any for Notch ligands is not well-defined (15, 19).

In this report, we present data demonstrating CD28 mediated NFκB signaling drives expression of Notch ligands DLL1, DLL4, and JAG1 on CD4 T cells within early hours of T cell activation. In contrast, signaling solely through TCR suppressed ligand expression on T cells, which is distinct from TCR dependent Notch activation. These data support a model whereby CD28 mediated signaling upregulates Notch ligand expression and subsequently these ligands associate in *cis* with Notch. In several other developmental systems in both invertebrates and vertebrates, *cis*-interaction between Notch and Notch ligands result in inhibition of Notch activity. We suggest in CD4 T cells, the *cis*-interaction between Notch receptor and Notch ligand is inhibitory and provides a mechanism to limit the duration or intensity of the TCR-induced Notch signal. Furthermore, when Notch ligand expression is blocked, *cis*-inhibition is relieved, thus driving Notch activation.

## Materials and Methods

### Mice

All animals were housed in animal facilities as per the guidelines approved by the Institutional Animal Care and Use Committee at the University of Massachusetts Amherst. C57BL/6J mice and C57BL/6-Tg(Tcra2D2, Tcrb2D2)1Kuch/J were purchased from the Jackson Laboratory (Bar Harbor, ME, USA). Spleens of C57BL/6-Tg(Nr4a1-EGFP/cre)820Khog/J reporter mice were obtained from Dr. Leonid Pobezinsky of the University of Massachusetts Amherst, Amherst, MA and Dr. Eric Huseby of University of Massachusetts Medical School, Worcester, MA. Mice aged 7–12 weeks were used for all experiments.

### T Cell Isolation and *in-vitro* Assays

CD4 T cells were isolated by magnetic separation using anti-CD4 magnetic particles (BD Pharmingen). Cells were activated after isolation with soluble anti-CD3ε (145-2C11) and anti-CD28 (clone 37.51) (BD Pharmingen) 1 μg/mL each, crosslinked with anti-hamster IgG (Sigma) 4.5 μL/mL. Cells were activated at 1.5 × 10^6^ cells/mL. Cells were activated in a 1:1 mixture of RPMI and DMEM (RDG) supplemented with 10% Fetal Bovine Serum (PEAK), L-Glutamine, Na-Pyruvate, Penicillin/Streptomycin, and 2-mercaptoethanol.

### BMDC and T Cell Co-culture

Bone marrow was collected from the femurs and tibias of female C57BL/6J mice. Cells cultured in RPMI-1640 medium supplemented with 10% Fetal Bovine Serum (PEAK), L-Glutamine, Na-Pyruvate, Penicillin/Streptomycin, and 2-mercaptoethanol in a 100 mm bacteriological petridish. The cells were then grown for 10 days in the presence of 200 U/mL of rmGM-CSF, with change of media on day 3, 6, and 8. After 10 days non-adherent cells in suspension were harvested and resuspended into RPMI complete with 10 ng/mL rmIL-4 (Biolegend) and 200 U/mL rmGM-CSF (Biolegend), plated at 1 × 10^6^ cells in a 12 well-tissue culture grade plate. One microgram per milliliter of LPS was added per well for LPS maturation of BMDCs. After 18 h cells were harvested stained with cell trace violet dye (Life Technologies) and pulsed with 10 μg/mL of MOG_35−55_ in a 24 well-plate for 2 h. Control BMDCs did not receive any MOG_35−55_ treatment. CD4 T cells isolated from 2D2 Transgenic mice were stained with CFSE (Life technologies). T cells were plated in a 48 well-tissue culture grade plate along with antigen pulsed BMDCs at a ratio of 10:1 (3 × 10^6^ T cells: 3 × 10^5^ BMDCs). Activation was conducted for indicated time points.

### Decoys for Notch Ligands

HEK 293T grown in 1:1 mixture of RPMI and DMEM supplemented with 10% Fetal Bovine Serum(GIBCO), l-Glutamine, Na-Pyruvate, and Penicillin/Streptomycin, HEK 293 T cells were transiently transfected with rAAV-collagen-N1ECD or rAAV-collagen constructs were made by Dr. Yong Ran and were obtained from Dr. Todd E. Golde at the University of Florida. Supernatants collected from the transfected cells and concentrated using Amicon Ultra Centrifugal filter units (Millipore) as described.

### Flow Cytometry and AMNIS Imaging Flow Cytometry

Surface staining of T cells was performed with 1% BSA in PBS using indicated antibodies CD25-APC, DLL1-APC (HMD1–3), DLL4-APC (HMD4–1), DLL4-PE (HMD4–1), CD339 (JAG1)-APC (HMJ1–29), CD339(JAG1)-PE (HMJ1–29) (Biolegend), Notch1-PE (22E5) (eBioscience). Intracellular staining was performed for detecting intracellular Notch using Foxp3 staining buffer set (eBioscience) for fixing and permeabilizing the cells and staining with Notch1-PE (mN1A) antibody (BD Pharmingen). For live-dead staining Zombie violet fixable dye (Biolegend) was used prior to fixation. Flow cytometry data was acquired on a BD LSR Fortessa (5 Laser) and analyzed using FlowJo software after gating on live CD4^+^ T population. Imaging flow cytometry data was acquired on AMNIS ImageStream^X^ MkII and analyzed using IDEAS software.

### Confocal Imaging

Surface staining of T cells was performed using indicated antibodies DLL1-APC (HMD1–3), DLL4-APC (HMD4–1), JAG1-APC (HMJ1–29) (Biolegend), Notch1-PE (22E5) (eBioscience). For confocal microscopy, poly-d-lysine coated MatTek glass bottomed culture dishes were used with No. 1.5 cover glass on the bottom. Cells were first surface stained for the ligands and the Notch1ECD followed by fixation with 4% PFA/PBS for 10 min at room temperature, quenched with 50 mM MNH_4_Cl/PBS for 5 min. Fixed cells were then incubated with 300 nM DAPI for 20 mins at RT. Cells were then washed three times with prewarmed PBS 3–4 min each and then 200 μL PBS was added to the culture dishes and cells were imaged using Nikon A1R-SIMe. Images were analyzed using NIS elements software.

### Inhibitors

The inhibitors used for the experiments are Lck inhibitor (CAS 213743-31-8) (Sigma), PI3K inhibitor LY294002 (CST), Akt inhibitor MK-2206(Sigma), and NFκB inhibitor BAy11-7085 (Sigma).

### Statistical Analysis

All data are represented as mean ± SEM. Statistical Analysis was performed using the GraphPad Prism5 software. *P*-values were determined using a two-tailed Student's *t*-test as indicated on the figure legends. A *p* < 0.05 was considered significant (^*^p < 0.05, ^**^p < 0.01, ^***^p < 0.001, ns, not significant). Each experiment was performed at least three times.

## Results

### T Cells Upregulate Notch Ligands Upon Activation

Activation of T cells by antibody cross-linking of the CD3ε chain of the TCR complex and CD28 leads to the activation of Notch1, through γ- secretase mediated cleavage and release of the Notch ICD (10–12). The potential mechanisms by which Notch is activated is linked to TCR mediated signaling in T cells (11, 15). Notch ligands, on the other hand, have been determined to be a decisive factor for Th polarization, based on the antigens encountered on the surface of antigen presenting cells (7, 27–29). In a purified population of T cells, whether Notch ligands are involved in Notch activation remains to be determined.

**Figure 1:**
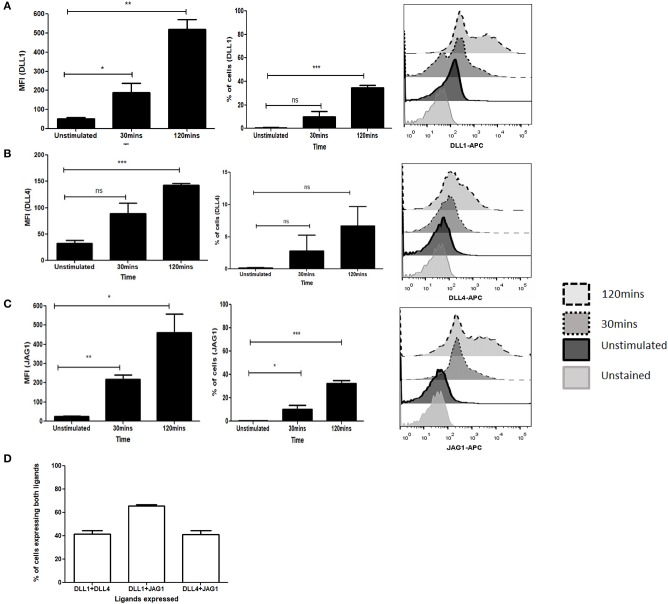
Activation of T cells induces expression of Notch ligands. CD4 T cells from C57BL/6 mice were treated with soluble anti-CD3ε and anti-CD28 at 1 μg/mL each for indicated time points. Cells were harvested and analyzed by flow cytometry by gating on live cell population as determined by absence of Zombie staining. MFI and percentage of ligand positive cells were plotted values were plotted for **(A)** DLL1, **(B)** DLL4, and **(C)** JAG1. Histograms to the right of **(A–C)** show expression of DLL1, DLL4, and JAG1 at each time point. **(D)** Percentage of cells showing co-expression of ligands on CD4 T cells. CD4 T cells from C57BL/6 mice were treated with soluble anti-CD3ε and anti-CD28 at 1 μg/mL each for 6 h and stained for antibodies against DLL1 and DLL4, or DLL1 and JAG1, or DLL4 and JAG1. Data represent three independent experiments. Data represent mean ± SEM. *p < 0.05, **p < 0.005, and ***p < 0.001. ns, not significant.

To determine the potential role of the Notch ligands DLL1, DLL4, and JAG1 in purified CD4 T cells we investigated the kinetics of Notch ligand expression following signaling through TCR and CD28. We observed an immediate upregulation of all ligands as early as 30 min following activation with soluble anti-CD3ε/anti-CD28 (Figures 1A–C, Figure S1A*)*. This expression is maintained even after 2 h of activation and shows a significant increase of ligands on the cell surface. Interestingly, not all the cells express individual Notch ligands, with about 40% of the cells expressing the ligands DLL1 and JAG1 by 2 h of activation (Figures 1A,C). In the case of DLL4, only about 10% ligand positive cells are observed at 2 h of activation (Figure 1B). This indicates that the kinetics of ligand expression varies between the ligands. Additionally, as shown in Figure 1D and Figures S1B–D CD4 T cells can express multiple ligands. For this experiment all antibodies were tested on CHO-cells expressing Notch ligands DLL1 or JAG1 (Figure S11). Collectively the data shows that CD4 T cells are capable of expressing multiple Notch ligands early after activation with soluble anti-CD3ε/anti-CD28.

### Notch Ligands Expressed on T Cells Do Not Induce Notch Activation

**Figure 2:**
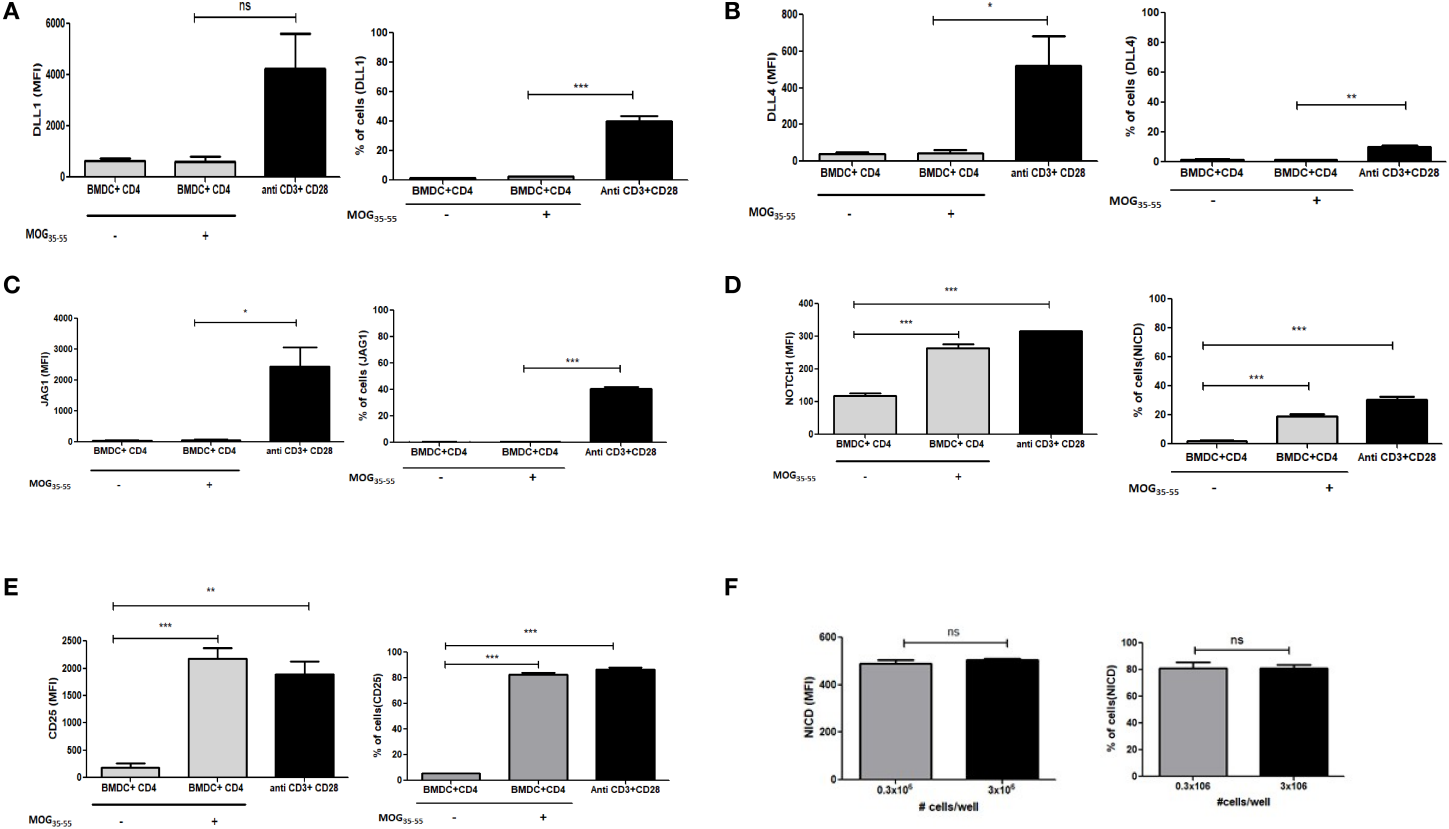
Notch ligands are not expressed on CD4 T cells in the presence of APCs but Notch1 is activated. CD4 T cells from 2D2 Transgenic mice were cocultured with MOG_35−55_ pulsed mature bone marrow derived dendritic cells (BMDC) obtained from 2D2 mice. T cells were stained with CFSE and BMDCs with Cell trace violet cell tracker dye before setting up for coculture. CD4 T cells stimulated with anti CD3ε or anti CD28 or anti CD3ε and anti-CD28, at 1 μg/mL each was activated along with the coculture. Cells harvested after 6 h and analyzed by flow cytometry for ligands and N1ICD and after 24 h for CD25. Analysis is done by gating on CFSE positive T cells and cell trace violet positive BMDCs. MFI values and percentage of positive cells for the indicated proteins for indicated treatments were plotted for **(A)** DLL1, **(B)** DLL4, **(C)** JAG1, **(D)** Intracellular cleaved Notch1 (N1ICD), and **(E)** CD25 on CD4 T cells. **(F)** MFI values and percentage of positive cells with intracellular Notch in CD4 T cells after 24 h of activation. CD4 T cells from C57BL/6 mice were stimulated with soluble anti-CD3ε and anti-CD28 at 1 μg/mL at a concentration of 0.3 × 10^6^ or 3 × 10^6^ for 24 h. Cells were harvested after 24 h and intracellularly stained for Notch1 ICD. Data represent three independent experiments. Data represent mean ± SEM. *p < 0.05, **p < 0.005, and ***p < 0.001. ns, not significant.

Data from earlier studies suggested that expression of different ligands on the APCs are important regulators of T helper cell subset formation. However, the specific status or role of ligand expression on the T cells in the presence of APCs has not been investigated. To determine if Notch ligands are also expressed in the presence of APCs on the T cells, we generated bone marrow derived dendritic cells (BMDCs) and co-cultured the BMDCs with T cells. Specifically, CD4 T cells derived from transgenic 2D2 mice, capable of recognizing MOG_35−55_ peptide, were activated in the presence of MOG_35−55_ pulsed BMDCs. 2D2 T cells were also activated with soluble antibodies against CD3ε and CD28 together in the absence of any APC or antigen. We did not observe an increase in any of the three Notch ligands on the T cells activated with BMDCs (Figures 2A–C and Figures S2A–D). As we have shown previously (10, 11) T cells activated with antibodies against CD3ε and CD28 together showed increase in activated Notch (Figure 2D). Additionally, the level of activated Notch in T cells stimulated with anti-CD3ε/anti-CD28, was comparable to the level found in T cells stimulated with MOG_35−55_ pulsed BMDCs (Figure 2D). The activation status of the CD4 T cells under different conditions was confirmed by the level of CD25 expression (Figure 2E). Because the production of NICD (Figure 2D) and the expression of CD25 (Figure 2E) are both known outcomes of Notch activation, these data also provide a measure of Notch activity, although it is likely that other factors also contribute to CD25 expression. In order to understand if T cells adjacent to each other can act as ligand presenting cell to the neighboring cell, we activated CD4 T cells at different concentrations in the absence of APC. So that diluting the cells in the culture medium will reduce cell-to-cell contact, reducing the interactions between Notch ligand and receptor between two adjacent T cells if any. However, activation of CD4 T cells at two different concentrations did not show any changes in activation of Notch, even after 10 fold increase in the number of cells for activation. Thus activation of Notch is occurring independent of trans-presentation of ligand to Notch (Figure 2F and Figure S3). Thus, as observed previously, Notch is also activated in the absence of ligands on the T cells. Signaling through TCR in combination with CD28 is sufficient to activate Notch in mature CD4 T cells (10, 11, 15). In contrast to what we observed in CD4 purified T cells activated by soluble anti-CD3ε/anti-CD28, APCs + antigen do not induce ligand expression on CD4 T cells (Figures 2A–C). These data demonstrate that APC presentation of antigen to CD4 T cells does not induce ligand expression on the CD4 T cells. However, even though the CD4 cells do not express Notch ligands, robust Notch activation is induced in these cells.

### Notch Ligands Colocalize With Receptors on the Same T Cells and Can Inhibit Activated Notch by *cis*-Interaction

**Figure 3:**
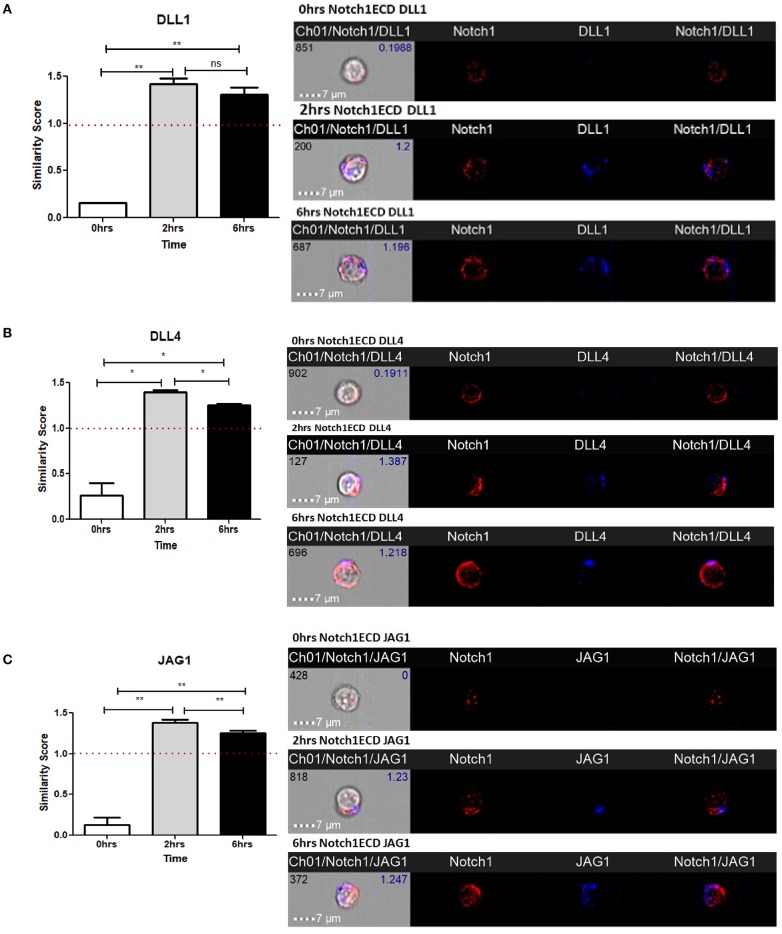
Notch ligands expressed on CD4 T cells colocalize with Notch receptor on the same cell. CD4 T cells from C57BL/6 mice were treated with soluble anti-CD3ε and anti-CD28 at 1 μg/mL for indicated time points. Cells were harvested and analyzed by AMNIS Imaging flow cytometry. Similarity scores indicate the degree of colocalization of each ligand with Notch extracellular domain. Similarity score values were plotted along with images acquired 60× magnification for **(A)** DLL1 and N1ECD, **(B)** DLL4 and N1ECD, and **(C)** JAG1 and N1ECD. Images to the right of **(A–C)** show expression and colocalization of each of DLL1, DLL4, JAG1 with N1ECD at time point. Data represent three independent experiments. The analysis was done by gating on live cells as determined by absence of zombie staining. Data represent mean ± SEM. *p < 0.05, **p < 0.005. ns, not significant.

**Figure 4:**
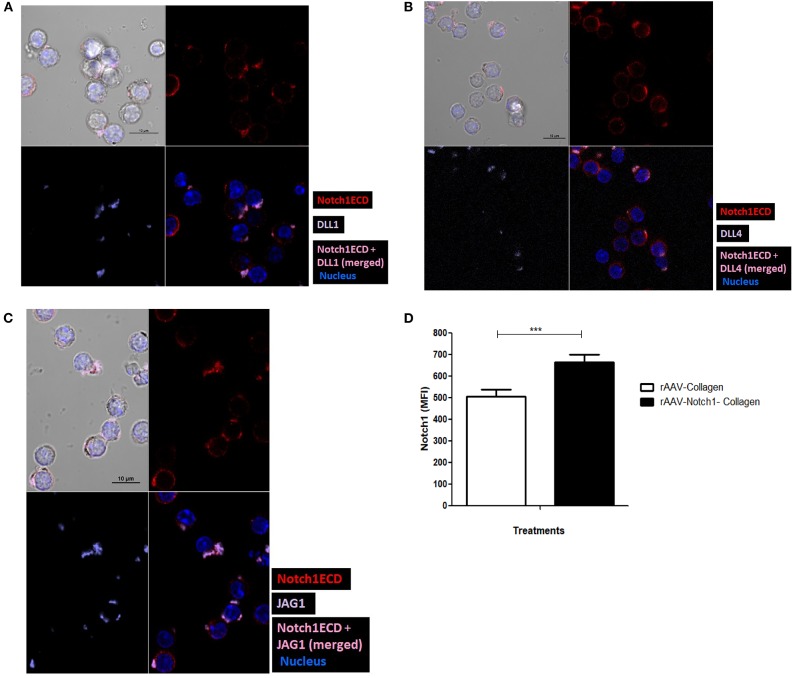
Notch ligands expressed on CD4 T cell *cis*-inhibit activated Notch upon colocalization. CD4 T cells from C57BL/6 mice were treated with soluble anti-CD3ε and anti-CD28 at 1 μg/mL for 6 h and surface stained for ligands (purple), Notch1ECD (red), and for nucleus cells were treated with DAPI. Colocalization of each of the ligands **(A)** DLL1, **(B)** DLL4, and **(C)** JAG1 with N1ECD at indicated time points. **(D)** Conditioned media containing decoy peptides collected from HEK293 T cells transiently transfected with rAAV-collagen-Notch1ECD or rAAV-collagen. CD4 T cells activated with 1 μg/mL of each of soluble anti-CD3ε and anti-CD28 in the presence of indicated amount of the concentrated conditioned media for 24 h. Bar graphs MFI of N1ICD for each treatment. Data represent three independent experiments. The analysis was done by gating on live cells as determined by absence of zombie staining. Data represent mean ± SEM. ***p < 0.001.

As shown in our previous experiments, Notch is activated solely upon signaling through CD3ε and CD28. However, it is still unclear whether Notch ligands expressed on CD4 T cells regulate Notch activity. Thus, we wanted to investigate the physiological role of ligands on T cells. Previous studies in *Drosophila* have shown that the interaction between the Notch receptor and the ligand can occur either in *cis* or in *trans*. When membrane bound ligands activate the Notch receptor on a neighboring cell, the phenomenon is called *trans*-interaction (1, 19, 34). However, when Notch ligands and receptors interact on the same cell, this sends an inhibitory signal and thus suppresses intracellular signaling of Notch. This phenomenon is known as *cis*-interaction or *cis*-inhibition (35, 36). *Cis*-inhibition has been shown to be an important player in determining fates of different types of cells in the developmental process of *Drosophila*, such as neuro-genesis, wing margin formation, and also in the maintenance of postnatal human epidermal stem cells (37, 38). Whether such a mechanism exists in mature CD4 T cells to regulate Notch1 activity is unknown. To study *cis* interaction, we activated T cells for 2 and 6 h and imaged the cells by AMNIS Imaging Flow Cytometry to assess colocalization of Notch extracellular domain with the ligands. For these experiments, we used an antibody specifically recognizing the extracellular domain of Notch1 (N1-ECD), in addition to antibodies directed against the DLL1, DLL4, and JAG1. The unstimulated cells showed poor or no expression of Notch ligands and only expressed low levels of N1ECD on the surface (Figures 3A–C). An increase in expression of all three Notch ligands was observed after 2 and 6 h of activation. In addition, surface staining for the ligands and N1ECD demonstrated colocalization and interaction in *cis* (Figures 3A–C). The degree of colocalization, as measured by the calculated similarity score, shows that the N1ECD and ligands colocalize at 2 and 6 h and there is no expression of ligand on the cell surface in unstimulated cells. This supports the previous data obtained by flow cytometry (Figures 1A–C). The cells display surface expression of N1ECD which increases with the activation of the T cells as well. Interestingly, unlike N1ECD, the ligands are concentrated at certain positions on the cell surface. We employed confocal microscopy to further confirm the colocalization we observed in our AMNIS data. In the images shown in Figures 4A–C, our confocal data demonstrate that ligands are always associated with N1ECD.

Since we have observed earlier that Notch activation can occur independent of Notch ligands on the CD4 T cells (Figure 2D), we next wanted to address the exact role played by the ligands in Notch activation. Studies in vertebrates and invertebrates show that *cis*-interaction of the ligands and receptor can inhibit the activation of Notch (35, 36). One such DSL ligand DLL3 has been shown to be an antagonist of Notch signaling (39). In order to determine whether *cis*-inhibition occurs in T cells, we used decoys mimicking the extracellular domain of Notch, which can block ligand-receptor interaction by binding to the Notch ligands, to understand the effect of same cell interactions of Notch receptor and ligand. For these experiments, we transfected HEK293T cells with the soluble N1-Col-rAAV and Col-rAAV constructs and collected the supernatants. We used these supernatants containing decoy N1 ECD to determine if it blocks Notch receptor-ligand interaction. The decoy containing supernatants were concentrated and added along with anti-CD3ε/anti-CD28 CD4 T cells. We observed a significant increase in activated Notch in CD4 T cells activated for 24 h, upon blocking receptor-ligand interaction with the Notch decoy (Figure 4D). This suggests that *cis*-inhibition by Notch ligands may regulate Notch activity in T cells. The decoys likely prevent *cis*-interaction between Notch ligand and receptor which, in turn, results in Notch activation. Thus, from these above data, we concluded that Notch ligands, when expressed on CD4 T cells, are closely associated with N1ECD and this may result in *cis*-inhibition of Notch activity although further experimentation, such as FRET, is required to conclude direct physical interaction between Notch and ligands and mutational analysis of sites of interaction could allow us to determine whether this interaction results in cis-inhibition of Notch.

### Signaling Through CD28 Is Sufficient to Induce Notch Ligand Expression and CD3 Signals Suppress Ligand Expression on the CD4 T Cells

**Figure 5:**
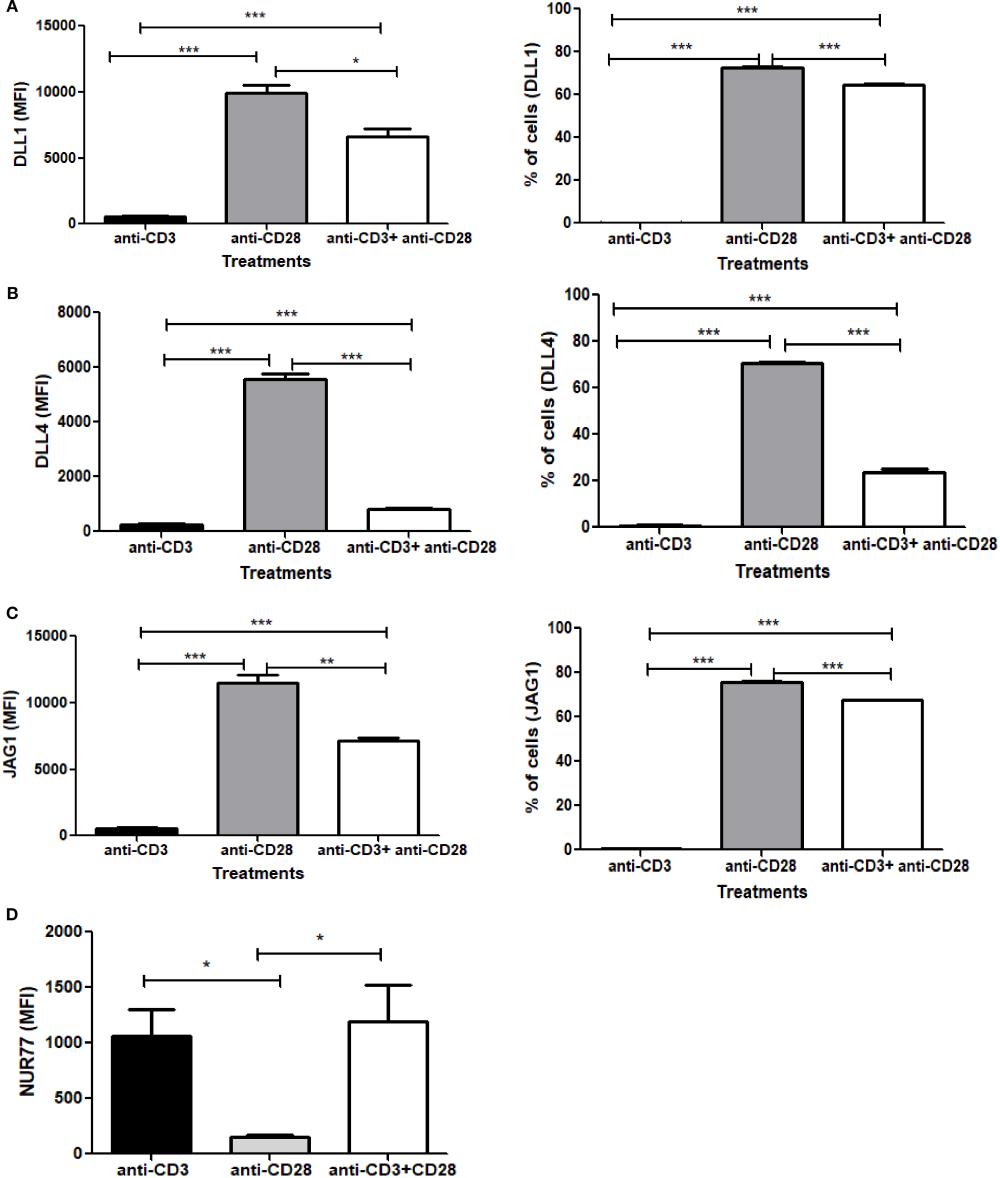
Signaling through CD28 is sufficient to induce expression of Notch ligands on CD4 T cells and signaling through CD3 suppresses ligand expression. CD4 T cells from C57BL/6 mice were treated with soluble anti-CD3ε alone or anti-CD28 alone or anti-CD3ε and anti-CD28 for 6 h. Cells were harvested and analyzed by flow cytometry by gating on live cell population as determined by absence of Zombie staining. MFI values and percentage of ligand positive cells were plotted for **(A)** DLL1, **(B)** DLL4, and **(C)** JAG1. **(D)** CD4 T cells from Nur77-GFP reporter mice were treated with either soluble anti-CD3ε or anti-CD28 or anti-CD28 and anti-CD3ε together for 6 h. Cells were harvested and analyzed by flow cytometry by gating on live cell population as determined by the absence of Zombie staining. Data represents three independent experiments Data represents mean ± SEM. *p < 0.05, **p < 0.005, and ***p < 0.001.

Signaling through TCR and CD28 is well-known to activate Notch, however, the contribution of TCR and/or CD28 to Notch ligand expression has not been explored prior to the data reported here. Since we observed an induction of Notch ligands by combined CD3ε and CD28 signaling, we wanted to explore how individual signaling through CD3ε and CD28 contribute to the process of Notch ligand expression on CD4 T cells. We stimulated CD4 T cells with antibodies against CD3ε alone, CD28 alone or a combination of antibodies against CD3ε and CD28 for 6 h, followed by surface staining for DLL1, DLL4, and JAG1 Surprisingly signaling through CD28 alone was sufficient to upregulate all three ligands on the cells (Figures 5A–C). These data indicate that signaling solely through CD28 is sufficient to induce Notch ligand expression on CD4 T cells. This is in contrast to the induction of Notch activation where signaling through CD28 does not activate Notch but signals through CD3ε alone are sufficient to induce Notch activation in primary CD4 T cells (10).

As shown above, CD28 signaling alone can induce robust Notch ligand expression on naive CD4 T cells. However, when we compared ligand expression induced by signals through CD28 to ligand expressed induced by the combination of anti-CD3ε/anti-CD28, significantly lower levels of ligand expression are induced by anti-CD3ε/anti-CD28 (Figures 5A–C). These data led us to consider that signaling through CD3ε may suppress ligand expression. Signaling through CD28 alone is not capable of activating T cells. Signaling through CD3ε alone is not sufficient to completely activate T cells either so we wanted to assess the relative contribution of CD3 and CD28 to TCR signal strength. For that, we used Nur77-GFP reporter mice (40). Nur77 is an immediate-early gene that is upregulated by TCR signaling in thymocytes as well as mature T cells (41). In this system, GFP expression, driven by the Nur77 promoter, acts as an indicator of the magnitude of the strength of each signal through CD3ε or CD28 or the combination of the two together (42). Thus, CD4 T cells obtained from these mice were activated with either CD3ε alone or CD28 alone or CD3ε plus CD28 together for 6 h, the magnitude of activation was measured by the increase in GFP expression. We observed that signaling through CD3ε induces significant GFP expression. However, no measurable Nur77 is detected in cells stimulated with anti-CD28 (Figure 5D). Thus, signaling solely through CD28 is correlated with the highest levels of ligand expression on T cells, whereas signal through CD3ε as measured by Nur77 -GFP expression partially suppresses ligand expression (Figures 5A–D and Figures S4, S5). However, once ligands are induced through CD28 signaling, CD3ε signals partially suppress the percent of cells expressing Notch ligands and significantly decrease the level of ligand expression as measured by MFI (Figures S4A–C). Thus, CD28 acts as an inducer of Notch ligands in CD4 T cells in contrast to CD3ε, which suppresses Notch ligand expression.

**Figure 6:**
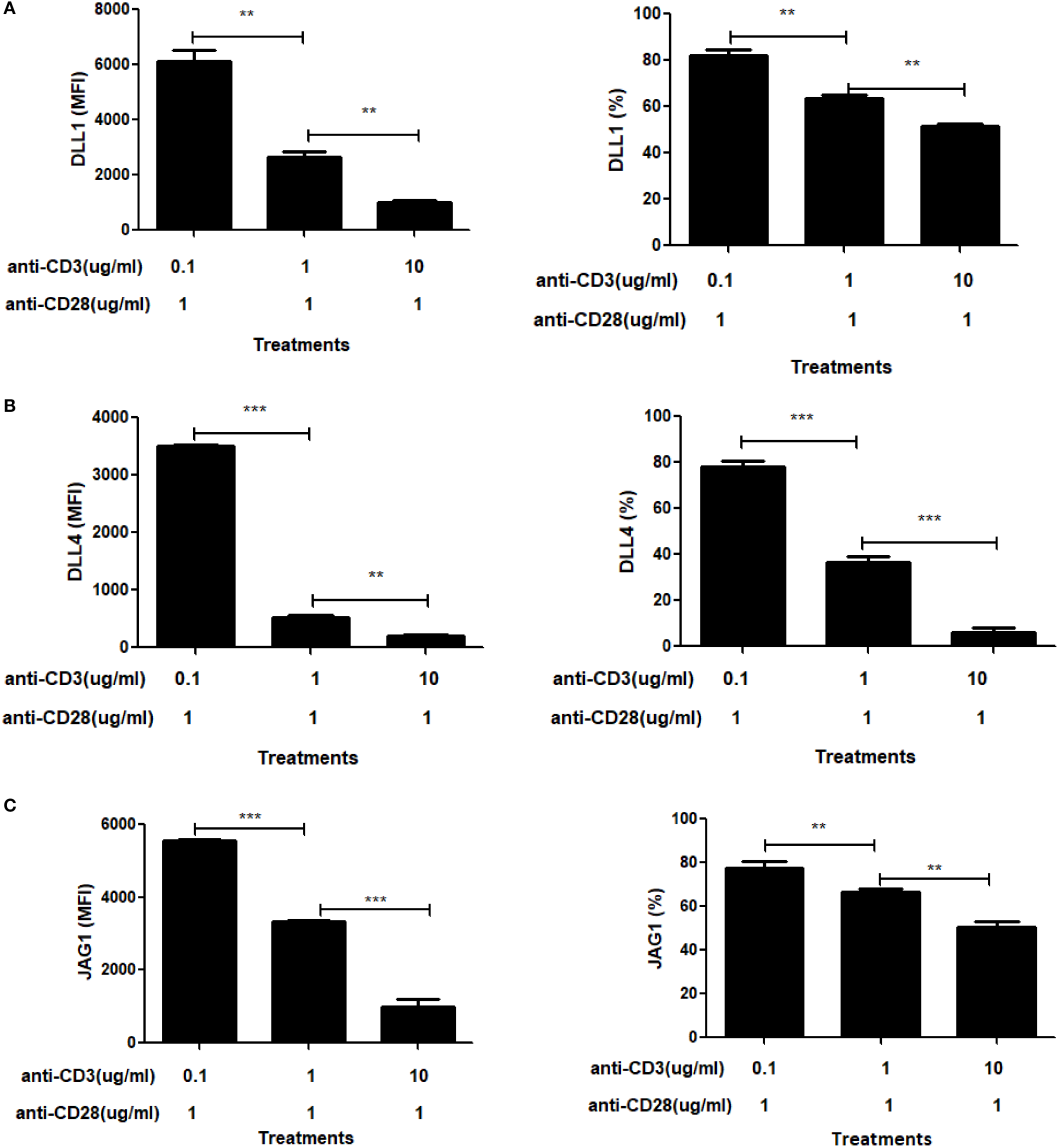
Signal through CD3 shows a dose dependent suppressive effect on Notch ligand expression. CD4 T cells from C57BL/6 mice were stimulated with the indicated concentrations of anti-CD3+ and 1 mg/mL of anti-CD28 for 6 h. Cells were harvested and analyzed by flow cytometry by gating on live cell population as determined by absence of Zombie staining. MFI values and percentage of ligand positive cells were plotted for **(A)** DLL1, **(B)** DLL4, and **(C)** JAG1. Histograms to the right of **(A–C)** show expression of DLL1, DLL4, and JAG1 with indicated treatments. Data represents three independent experiments MFI value. Data represent mean ± SEM. **p < 0.005 and ***p < 0.001.

To further explore the role of CD3ε on ligand expression, we titered the dose of soluble anti-CD3ε while keeping the dose of anti-CD28 constant (Figures 6A–C). In these experiments, we found that at limiting doses of anti-CD3ε (0.1 μg/mL), all three ligands were expressed at high levels whereas at 10 and 100-fold increases in anti-CD3ε (1 and 10 μg/mL), ligand levels dropped significantly. Both the percentage of CD4 T cells expressing ligands as well as the MFI of ligand expression diminished significantly in a dose-dependent fashion. These data indicate that increasing signals through TCR result in suppression of ligand expression.

### NFκB Regulates Notch Ligand Expression Downstream of CD28

CD28 signaling is known to regulate multiple proteins and signaling pathways, such as PI3K/PKB and NFAT, differently than TCR signaling (43–47). One such target of CD28 are the NFκB family proteins. The IL-2 promoter has two NFκB binding sites which are dependent on CD28 and are known as the CD28 response elements (CD28RE) (48–52). NFκB is sequestered in the cytoplasm by IκB and phosphorylation of IκB by IκB kinase (IKK) targets it for degradation, allowing NF-κB to enter the nucleus. CD28 signaling alone increases the activity of IKK and thus aids in the translocation of NFκB to the nucleus (53–55). In primary macrophages, JAG1 is induced in an NFκB dependent fashion (56). Additionally, in human Jurkat T cells, *Jag1* mRNA increases almost 4-fold upon activation with PMA which activates NFκB signaling (57).

**Figure 7:**
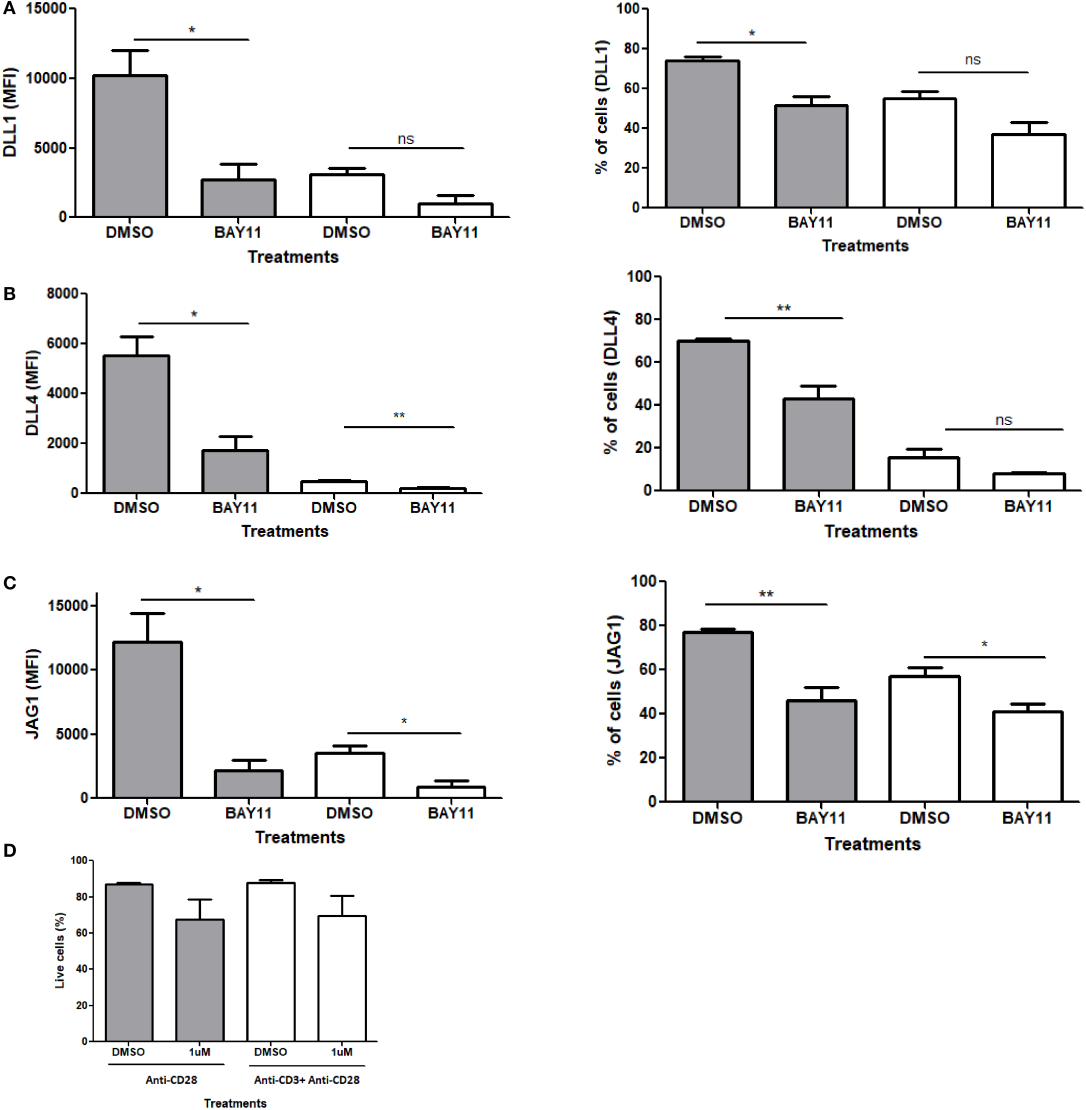
NFκB regulates Notch ligand expression downstream of CD28. CD4 T cells from C57BL/6 mice were treated with soluble anti CD28 or anti-CD3ε and anti-CD28 for 6 h in the presence of NFκB inhibitor BAy11 at 1 μM or DMSO as control. Cells were harvested and analyzed by flow cytometry by gating on live cell population as determined by the absence of Zombie staining. MFI values and percentage of ligand positive cells were plotted for **(A)** DLL1, **(B)** DLL4, and **(C)** JAG1. **(D)** Viability of cells post-treatment. Cells were harvested and analyzed by flow cytometry by gating on live cell population as determined by the absence of Zombie staining. Data represents three independent experiments Data represents mean ± SEM. *p < 0.05, **p < 0.005. ns, not significant.

Based on the evidence above, we examined the role of NFκB in CD28 mediated Notch ligand expression. For these experiments, we used a pharmacological inhibitor of NFκB, BAy11-7082, which blocks the translocation of NFκB to the nucleus. Naïve CD4 T cells were stimulated with antibodies against CD28 or CD3ε plus CD28 in the presence or absence of the inhibitor. Unlike the other potential targets of CD28 (Figures S6–S8), the inhibition of NFκB showed a significant decrease in Notch ligand expression upon stimulation with anti-CD28 alone (Figures 7A–C, Figure S9). To control for loss of ligand expression due to potential toxicity of BAY11, viability of cells treated with BAY11 as compared to DMSO vehicle was assessed and no significant toxicity was observed with BAY11 treatment (Figure 7D). Therefore, we conclude that signals through CD28 and NFκB are, at least in part, responsible for Notch ligand expression on CD4 T cells.

## Discussion

Although ligand independent Notch activation previously has been described in different systems, the involvement of Notch ligands in the Notch activation process in T cells has not been well-studied (10, 11, 15). The role of Notch ligands in the activation of Notch signaling in T cells has been debated for several years. Early data from our lab and others (10, 11) suggested that, in T cells, Notch activation may occur through ligand independent events. Indeed, recent work from Winandy and Colleagues, provide compelling evidence for ligand independent activation of Notch in CD4 T cells (15). In this report, we demonstrate that even though they do not appear to participate in Notch activation, ligands are induced by signals through CD28 and co-localize with Notch. Our data suggest the *cis*-interaction between Notch and Notch ligands results in blockade of Notch activation. Furthermore, we provide evidence that engagement with the TCR complex results in a significant diminution of ligand expression on CD4 T cells, potentially relieving *cis*-inhibition of Notch activity.

We initiated our study with a question addressing the status of the three Notch ligands DLL1, DLL4, and JAG1 on CD4 T cells, post-activation. In previous studies, these three ligands were shown to be the main player in modulating Notch signals and determining T helper cell fate (7, 27–29). Surprisingly, we found considerable change in the levels of surface expression of these three Notch ligands on mature CD4 T cells, within an hour of activation. This expression increases with progression of the activated state of the T cells. Other data reported DLL1 expression in activated T cells, but these experiments examined ligand expression several days following TCR activation (58). In contrast, our study shows that Notch ligands may be expressed on T cells very early in the activation process (Figures 1A–C). Furthermore, ligand expression is exclusively CD28 signaling dependent. The complete absence of ligand expression by CD3ε mediated signaling alone, suggests that CD3ε may act as a negative regulator of ligand expression (Figures 5A–C). This also is supported by the reduction in overall ligand expression upon activation using a combined CD3 and CD28 signaling. All three ligands showed the same trend in expression patterns and appeared to be regulated by a similar mechanism, although the kinetics of individual ligand expression can vary. Also, based on the transcript data, we concluded that the ligand transcripts do not show any signs of regulation by CD3 or CD28 signaling, hence indicative of a post-transcriptional regulation (Figure S10).

Activation of CD4 T cells with MOG_35−55_ peptide presenting BMDCs does not induce ligand expression on T cells. Nevertheless, in the absence of ligand on T cells, there was a considerable level of activated Notch observed in CD4 T cells. This was comparable to the amount of activated Notch in CD4 T cells undergoing activation with soluble antibodies against CD3ε and CD28. Thus, the results obtained from this experiment strongly suggest that ligand expression on mature T cells only occurs in the absence of APCs and in case of antigen independent activation of T cells. This *cis*-inhibition process may represent a fail-safe against antigen-independent T cell activation or a feedback response in case of particularly intense or prolonged co-stimulatory signals. Furthermore, it will be interesting to study the differences in the strength of signal sent through APC mediated antigen dependent vs. antigen independent, antibody mediated activation. Whether or not the presence or absence of antigen during T cell activation causes any changes in the magnitude of signal, remains unaddressed. Our observation reestablishes the fact that Notch activation in mature CD4 T cells is ligand independent and regulated by TCR signaling only (10, 11, 15).

In order to further understand the impact of ligand expression on T cells, we wished to examine whether there is physical interaction between the ligands and the receptors on the same cells. We used AMNIS Imagestream analysis and confocal microscopy for these studies which demonstrated colocalization of ligand and receptor on the cell membrane. Since AMNIS is also an excellent tool to quantify the extent of colocalization and the number of cells showing such phenomenon, this approach provided us with a quantitative view of Notch/Notch ligand co-localization on a single cell basis. The data demonstrated that Notch ligands and Notch1 receptor colocalize on activated CD4 T cells (Figures 3A–C). The effect of such *cis*-interaction, when examined using decoy peptide, suggests that these ligands are capable of inhibiting Notch activation when interacting in *cis*. However, since our data demonstrate close localization between Notch and ligands and do not demonstrate direct contact between these molecules, further experiments are required to explain the phenomenon and confirm the occurrence of cis inhibition by the ligands. Moreover, it will be interesting to see the differences between the interaction of Notch with each type of ligand and their biological effects on CD4 T cells. Interestingly, unlike N1ECD, the ligands are concentrated at certain positions on the cell surface (Figures 4A–C). Whether or not, the punctate distribution of the ligands on the T cells surface found proximal to the CD28 engaged with an antibody, has not been determined.

Because we found ligand expression is a unique response to signaling through the CD28 costimulatory molecule, we attempted to define the signaling pathway downstream of CD28 responsible for ligand induction. Since both the Lck and PI3K-Akt pathways are activated by CD28 signaling, we used different inhibitors against the major components of these pathways. However, we found no indication that either Lck or PI3K/Akt plays a role in the induction of Notch ligand expression following treatment with anti-CD28 only (Figures S6–S8). In our experiments, we showed that CD3ε signaling resulted in the suppression of CD28 mediated induction of Notch ligand expression. Using Nur77-GFP reporter mice, we compared signal strength between CD3ε signaling alone to combined signaling through CD3ε and CD28. Here, we observed that the signal strength, as assessed by GFP expression, is similar in both the cases. This supports our observation that CD3ε signaling can suppress Notch ligand expression but cannot completely eliminate the induction of ligand expression induced by independent signaling through CD28 (Figures S4A–C). Thus, we conclude that Notch ligands are regulated by unique signaling through CD28 and signals through CD3ε likely is a negative regulatory factor for ligand expression. Furthermore, when individual pathways downstream of CD28 were tested as possible regulators of Notch ligands, we identified NFκB as a regulatory factor that contributes to ligand expression. Perhaps this is not surprising since in B cells, JAG1 has been shown to be regulated by NFκB (57). In case of T cells there are several pathways that lie immediately downstream of CD28 and act upon NFκB, such as PKCθ and Akt/mTOR. Therefore, it will be interesting to determine the intermediate signaling steps between CD28 and NFκB that regulate this pathway to induce Notch ligand expression.

**Figure 8:**
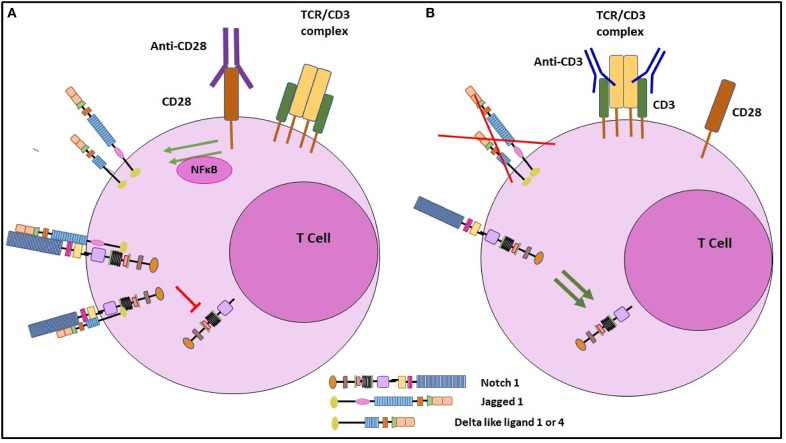
Schematic representation of the regulation of Notch ligand expression on CD4 T cells. **(A)** Signaling through CD28 by engagement with soluble anti CD28 antibody, induces Notch ligand expression on mature CD4 T cells. These ligands when *cis*-interact with Notch receptor, can inhibit cleavage of NICD, the active form of Notch. **(B)** Signaling through CD3ε inhibits Notch ligand expression on CD4 T cells. By inhibiting Notch ligand expression, CD3ε signaling prevents *cis* inhibition and thus induces Notch activation.

Our findings confirm previous findings of ligand independent activation of Notch in mature CD4 T cells (10, 11, 15). Additionally, we have defined a novel activity of CD28 in regulating Notch ligand expression. Also, these studies provide a basis for further understanding of the role of CD28 signaling in T cells. The colocalization of ligand and receptors on the same cell also suggests that there is a possibility of *cis*-inhibition of Notch activity in the T cells (Figure 8). We and others have shown previously that Notch activation contributes to the TCR signal strength (11, 33). It is interesting to note that our current data presented here reveal that Notch ligands are induced through CD28 engagement and the ligands co-localize with Notch suggesting that ligand expression may act through *cis*-inhibition to block Notch activity and, hence, prevent full T cell activation. Additionally, our data show that when signaling through CD28 is combined with signals through the TCR complex, ligand expression is significantly reduced in a dose dependent fashion and Notch activity is induced. This novel mechanism of CD28 mediated induction of Notch ligand on T cell surface provides an important insight to the significance of CD28 in impacting T cell function and opens an area of further study to understand *cis*-inhibition of Notch in mature CD4 T cells.

## Data Availability Statement

All datasets generated for this study are included in the article/Supplementary Material.

## Ethics Statement

This research was approved by the UMass IACUC committee.

## Author Contributions

AM performed most of the experiments and analyzed data. HS and KS performed experiments and analyzed data. AM, SS, HS, KS, RL, MC, JT, and BO designed experiments with contributions from LMM, TG, LM, and ST. YR synthesized the Notch decoy constructs. AM and BO conceived the study, supervised experimental design, and interpretation of data. AM and BO wrote the manuscript.

## Conflict of Interest

The authors declare that the research was conducted in the absence of any commercial or financial relationships that could be construed as a potential conflict of interest.

## References

[1] Artavanis-Tsakonas S Rand MD Lake RJ. Notch signaling: cell fate control and signal integration in development. Science. (1999) 284:770–6. 10.1126/science.284.5415.770 10221902

[2] Aster JC Pear WS Blacklow SC. The varied roles of notch in cancer. Annu Rev Pathol. (2017) 12:245–75. 10.1146/annurev-pathol-052016-100127 27959635 PMC5933931

[3] Osborne BA Minter LM. Notch signaling during peripheral T-cell activation and differentiation. Nat Rev Immunol. (2007) 7:64–75. 10.1038/nri1998 17170755

[4] Radtke F Fasnacht N MacDonald HR. Notch signaling in the immune system. Immunity. (2010) 32:14–27. 10.1016/j.immuni.2010.01.004 20152168

[5] Kopan R Ilagan MX. the canonical notch signaling pathway: unfolding the activation mechanism. Cell. (2009) 137:216–33. 10.1016/j.cell.2009.03.045 19379690 PMC2827930

[6] Tanigaki K Tsuji M Yamamoto N Han H Tsukada J Inoue H. Regulation of αβ/γδ T cell lineage commitment and peripheral T cell responses by Notch/RBP-J signaling. Immunity. (2004) 20:611–22. 10.1016/S1074-7613(04)00109-8 15142529

[7] Amsen D Blander JM Lee GR Tanigaki K Honjo T Flavell RA. Instruction of distinct CD4 T helper cell fates by different Notch ligands on antigen-presenting cells. Cell. (2004) 117:515–26. 10.1016/S0092-8674(04)00451-9 15137944

[8] Yamaguchi E Chiba S Kumano K Kunisato A Takahashi T Takahashi T. Expression of Notch ligands, Jagged1, 2 and Delta1 in antigen presenting cells in mice. Immunol Lett. (2002) 81:59–64. 10.1016/S0165-2478(01)00326-1 11841846

[9] Tanigaki K Han H Yamamoto N Tashiro K Ikegawa M Kuroda K. Notch-RBP-J signaling is involved in cell fate determination of marginal zone B cells. Nat Immunol. (2002) 3:443. 10.1038/ni793 11967543

[10] Palaga T Miele L Golde TE Osborne BA. TCR-mediated Notch signaling regulates proliferation and IFN-γ production in peripheral T cells. J Immunol. (2003) 171:3019–24. 10.4049/jimmunol.171.6.3019 12960327

[11] Dongre A Surampudi L Lawlor RG Fauq AH Miele L Golde TE. Non-canonical Notch signaling drives activation and differentiation of peripheral CD4^+^ T cells. Front Immunol. (2014) 5:54. 10.3389/fimmu.2014.00054 24611064 PMC3921607

[12] Adler SH Chiffoleau E Xu L Dalton NM Burg JM Wells AD. Notch signaling augments T cell responsiveness by enhancing CD25 expression. J Immunol. (2003) 171:2896–903. 10.4049/jimmunol.171.6.2896 12960312

[13] Guy CS Vignali KM Temirov J Bettini ML Overacre AE Smeltzer M. Distinct TCR signaling pathways drive proliferation and cytokine production in T cells. Nat Immunol. (2013) 14:262–70. 10.1038/ni.2538 23377202 PMC3577985

[14] Bheeshmachar G Purushotaman D Sade H Gunasekharan V Rangarajan A Sarin A. Evidence for a role for Notch signaling in the cytokine-dependent survival of activated T cells. J Immunol. (2006) 177:5041–50. 10.4049/jimmunol.177.8.5041 17015687

[15] Steinbuck MP Arakcheeva K Winandy S. Novel TCR-mediated mechanisms of Notch activation and signaling. J Immunol. (2018) 200:997–1007. 10.4049/jimmunol.1700070 29288204 PMC5854196

[16] Langridge PD Struhl G. Epsin-dependent ligand endocytosis activates Notch by force. Cell. (2018) 171:1383–96.e12. 10.1016/j.cell.2017.10.048 29195077 PMC6219616

[17] Nichols JT Miyamoto A Olsen SL D'Souza B Yao C Weinmaster G. DSL ligand endocytosis physically dissociates Notch1 heterodimers before activating proteolysis can occur. J Cell Biol. (2007) 176:445–58. 10.1083/jcb.200609014 17296795 PMC2063980

[18] Bozkulak EC Weinmaster G. Selective use of ADAM10 and ADAM17 in activation of Notch1 signaling. Mol Cell Biol. (2009) 29:5679–95. 10.1128/MCB.00406-09 19704010 PMC2772745

[19] Steinbuck MP Winandy S. A review of Notch processing with new insights into ligand-independent notch signaling in T-cells. Front Immunol. (2018) 9:1230. 10.3389/fimmu.2018.01230 29910816 PMC5992298

[20] Pui JC Allman D Xu L DeRocco S Karnell FG Bakkour S. Notch1 Expression in early lymphopoiesis influences B versus T lineage determination. Immunity. (1999) 11:299–308. 10.1016/S1074-7613(00)80105-3 10514008

[21] Dallas MH Varnum-Finney B Delaney C Kato K Bernstein ID. Density of the Notch ligand Delta1 determines generation of B and T cell precursors from hematopoietic stem cells. J Exp Med. (2005) 201:1361–6. 10.1084/jem.20042450 15851488 PMC2213184

[22] Rothenberg EV Ungerbäck J Champhekar A. Forging T-lymphocyte identity: intersecting networks of transcriptional control. Adv Immunol. (2016) 129:109–74. 10.1016/bs.ai.2015.09.002 26791859 PMC4747653

[23] Vijayaraghavan J Osborne BA. Notch and T cell function - a complex tale. Adv Exp Med Biol. (2018) 1066:339–54. 10.1007/978-3-319-89512-3_17 30030835

[24] Ciofani M Zúñiga-Pflücker JC. Notch promotes survival of pre-T cells at the beta-selection checkpoint by regulating cellular metabolism. Nat Immunol. (2005) 6:881–8. 10.1038/ni1234 16056227

[25] Fang TC1 Yashiro-Ohtani Y Del Bianco C Knoblock DM Blacklow SC Pear WS. Notch directly regulates Gata3 expression during T helper 2 cell differentiation. Immunity. (2007) 27:100–10. 10.1016/j.immuni.2007.04.018 17658278 PMC2801546

[26] Shin HM Tilahun ME Cho OH Chandiran K Kuksin CA Keerthivasan S. NOTCH1 can initiate NF-κB activation via cytosolic interactions with components of the T cell signalosome. Front Immunol. (2014) 5:249. 10.3389/fimmu.2014.00249 24904593 PMC4033603

[27] Maekawa Y Tsukumo S Chiba S Hirai H Hayashi Y Okada H. Delta1-Notch3 interactions bias the functional differentiation of activated CD4^+^ T cells. Immunity. (2003) 19:549–59. 10.1016/S1074-7613(03)00270-X 14563319

[28] Meng L Bai Z He S Mochizuki K Liu Y Purushe J. The Notch ligand DLL4 defines a capability of human dendritic cells in regulating Th1 and Th17 differentiation. J Immunol. (2016) 196:1070–80. 10.4049/jimmunol.1501310 26712946 PMC4930627

[29] Mukherjee S Schaller MA Neupane R Kunkel SL Lukacs NW. Regulation of T cell activation by Notch ligand, DLL4, promotes IL-17 production and Rorc activation. J Immunol. (2009) 182:7381–8. 10.4049/jimmunol.0804322 19494260 PMC2980695

[30] Constant S1 Pfeiffer C Woodard A Pasqualini T Bottomly K. Extent of T cell receptor ligation can determine the functional differentiation of naive CD4^+^ T cells. J Exp Med. (1995) 182:1591–6. 10.1084/jem.182.5.1591 7595230 PMC2192213

[31] Snook JP Kim C Williams MA. TCR signal strength controls the differentiation of CD4^+^ effector and memory T cells. Sci Immunol. (2018) 3:eaas9103. 10.1126/sciimmunol.aas9103 30030369 PMC6126666

[32] van Panhuys N Klauschen F Germain RN. T-cell-receptor-dependent signal intensity dominantly controls CD4(+) T cell polarization *in vivo*. Immunity. (2014) 41:63–74. 10.1016/j.immuni.2014.06.003 24981853 PMC4114069

[33] Izon DJ Punt JA Xu L Karnell FG Allman D Myung PS. Notch1 regulates maturation of CD4^+^ and CD8^+^ thymocytes by modulating TCR signal strength. Immunity. (2001) 14:253–64. 10.1016/S1074-7613(01)00107-8 11290335

[34] Bray SJ. Notch signalling in context. Nat Rev Mol Cell Biol. (2016) 17:722–35. 10.1038/nrm.2016.94 27507209

[35] del Álamo D Rouault H Schweisguth F. Mechanism and significance of *cis*-inhibition in Notch signalling. Curr Biol. (2011) 21:R40–7. 10.1016/j.cub.2010.10.034 21215938

[36] Sprinzak D Lakhanpal A Lebon L Santat LA Fontes ME Anderson GA. *Cis*-interactions between Notch and Delta generate mutually exclusive signalling states. Nature. (2010) 465:86–90. 10.1038/nature08959 20418862 PMC2886601

[37] Jacobsen TL Brennan K Arias AM Muskavitch MA. *Cis*-interactions between Delta and Notch modulate neurogenic signalling in Drosophila. Development. (1998) 125:4531–40. 9778511 10.1242/dev.125.22.4531

[38] Lowell S Jones P Le Roux I Dunne J Watt FM. Stimulation of human epidermal differentiation by delta-notch signalling at the boundaries of stem-cell clusters. Curr Biol. (2000) 10:491–500. 10.1016/S0960-9822(00)00451-6 10801437

[39] Ladi E Nichols JT Ge W Miyamoto A Yao C Yang LT. The divergent DSL ligand Dll3 does not activate Notch signaling but cell autonomously attenuates signaling induced by other DSL ligands. J Cell Biol. (2005) 170:983–92. 10.1083/jcb.200503113 16144902 PMC2171428

[40] Moran AE Holzapfel KL Xing Y Cunningham NR Maltzman JS Punt J. T cell receptor signal strength in Treg and iNKT cell development demonstrated by a novel fluorescent reporter mouse. J Exp Med. (2011) 208:1279–89. 10.1084/jem.20110308 21606508 PMC3173240

[41] Osborne BA Smith SW Liu ZG McLaughlin KA Grimm L Schwartz LM. Identification of genes induced during apoptosis in T lymphocytes. Immunol Rev. (1994) 142:301–20. 10.1111/j.1600-065X.1994.tb00894.x 7698798

[42] Au-Yeung BB Zikherman J Mueller JL Ashouri JF Matloubian M Cheng DA. A sharp T-cell antigen receptor signaling threshold for T-cell proliferation. Proc Natl Acad Sci U S A. (2014) 111:E3679–88. 10.1073/pnas.1413726111 25136127 PMC4156735

[43] Acuto O Michel F. CD28-mediated co-stimulation: a quantitative support for TCR signalling. Nat Rev Immunol. (2003) 3:939–51. 10.1038/nri1248 14647476

[44] Jenkins MK. The ups and downs of T cell costimulation. Immunity. (1994) 1:443–6. 10.1016/1074-7613(94)90086-8 7534615

[45] Rudd CE. Upstream-downstream: CD28 cosignaling pathways and T cell function. Immunity. (1996) 4:527–34. 10.1016/S1074-7613(00)80479-3 8673699

[46] Diehn M Alizadeh AA Rando OJ Liu CL Stankunas K Botstein D. Genomic expression programs and the integration of the CD28 co-stimulatory signal in T cell activation. Proc Natl Acad Sci U S A. (2002) 99:11796–801. 10.1073/pnas.092284399 12195013 PMC129348

[47] Appleman LJ van Puijenbroek AA Shu KM Nadler LM Boussiotis VA. CD28 co-stimulation mediates downregulation of p27kip1 and cell cycle progression by activation of the PI3K/PKB signaling pathway in primary human T cells. J Immunol. (2002) 168:2729–36. 10.4049/jimmunol.168.6.2729 11884439

[48] Rudd CE Schneider H. Unifying concepts in CD28, ICOS and CTLA4 co-receptor signalling. Nat Rev Immunol. (2003) 3:544–56 10.1038/nri1131 12876557

[49] Shapiro VS Truitt KE Imboden JB Weiss A. CD28 mediates transcriptional upregulation of the interleukin-2 (IL-2) promoter through a composite element containing the CD28RE and NF-IL-2B AP-1 sites. Mol Cell Biol. (1997) 17:4051–8. 10.1128/MCB.17.7.4051 9199340 PMC232258

[50] Lai JH Horvath G Subleski J Bruder J Ghosh P Tan TH. RelA is a potent transcriptional activator of the CD28 response element within the interleukin 2 promoter. Mol Cell Biol. (1995) 15:4260–71. 10.1128/MCB.15.8.4260 7623820 PMC230665

[51] Fraser JH Rincón M McCoy KD Le Gros G. CTLA4 ligation attenuates AP-1, NFAT and NF-kappaB activity in activated T cells. Eur J Immunol. (1999) 29:838–44. 10.1002/(SICI)1521-4141(199903)29:03<838::AID-IMMU838>3.0.CO;2-P 10092086

[52] Verweij CL Geerts M Aarden LA. Activation of interleukin-2 gene transcription via the T-cell surface molecule CD28 is mediated through an NF-κB-like response element. J Biol Chem. (1991) 266:14179–82. 10.1016/1043-4666(91)90175-D 1650350

[53] Bryan RG Li Y Lai JH Van M Rice NR Rich RR. Effect of CD28 signal transduction on c-Rel in human peripheral blood T cells. Mol Cell Biol. (1994) 14:7933–42. 10.1128/MCB.14.12.7933 7969133 PMC359332

[54] Lai JH Tan TH. CD28 signaling causes a sustained down-regulation of I kappa B alpha which can be prevented by the immunosuppressant rapamycin. J Biol Chem. (1994) 269:30077–80. 7982907

[55] Harhaj EW Good L Xiao G Uhlik M Cvijic ME Rivera-Walsh I. Somatic mutagenesis studies of NF-κB signaling in human T cells: evidence for an essential role of IKKγ in NF-κB activation by T-cell costimulatory signals and HTLV-I Tax protein. Oncogene. (2000) 19:1448–56. 10.1038/sj.onc.1203445 10723136

[56] Foldi J Chung AY Xu H Zhu J Outtz HH Kitajewski J. Autoamplification of Notch signaling in macrophages by TLR-induced and RBP-J-dependent induction of Jagged1. J Immunol. (2010) 185:5023–31. 10.4049/jimmunol.1001544 20870935 PMC3010732

[57] Bash J Zong WX Banga S Rivera A Ballard DW Ron Y. Rel/NF-κB can trigger the Notch signaling pathway by inducing the expression of Jagged1, a ligand for Notch receptors. EMBO J. (1999) 18:2803–11. 10.1093/emboj/18.10.2803 10329626 PMC1171361

[58] Furukawa T Ishifune C Tsukumo SI Hozumi K Maekawa Y Matsui N. Transmission of survival signals through Delta-like 1 on activated CD4^+^ T cells. Sci Rep. (2016) 6:33692. 10.1038/srep33692 27659682 PMC5034251

