# Supplementary material for: CD28 Signaling Drives Notch Ligand Expression on CD4 T Cells
Source: Front Immunol. 2020 May 7;11:735. doi: 10.3389/fimmu.2020.00735 (PMC7221189; doi:10.3389/fimmu.2020.00735)
Supplement: Supplementary file 1 [file Presentation_1.pptx]

## Slide 1
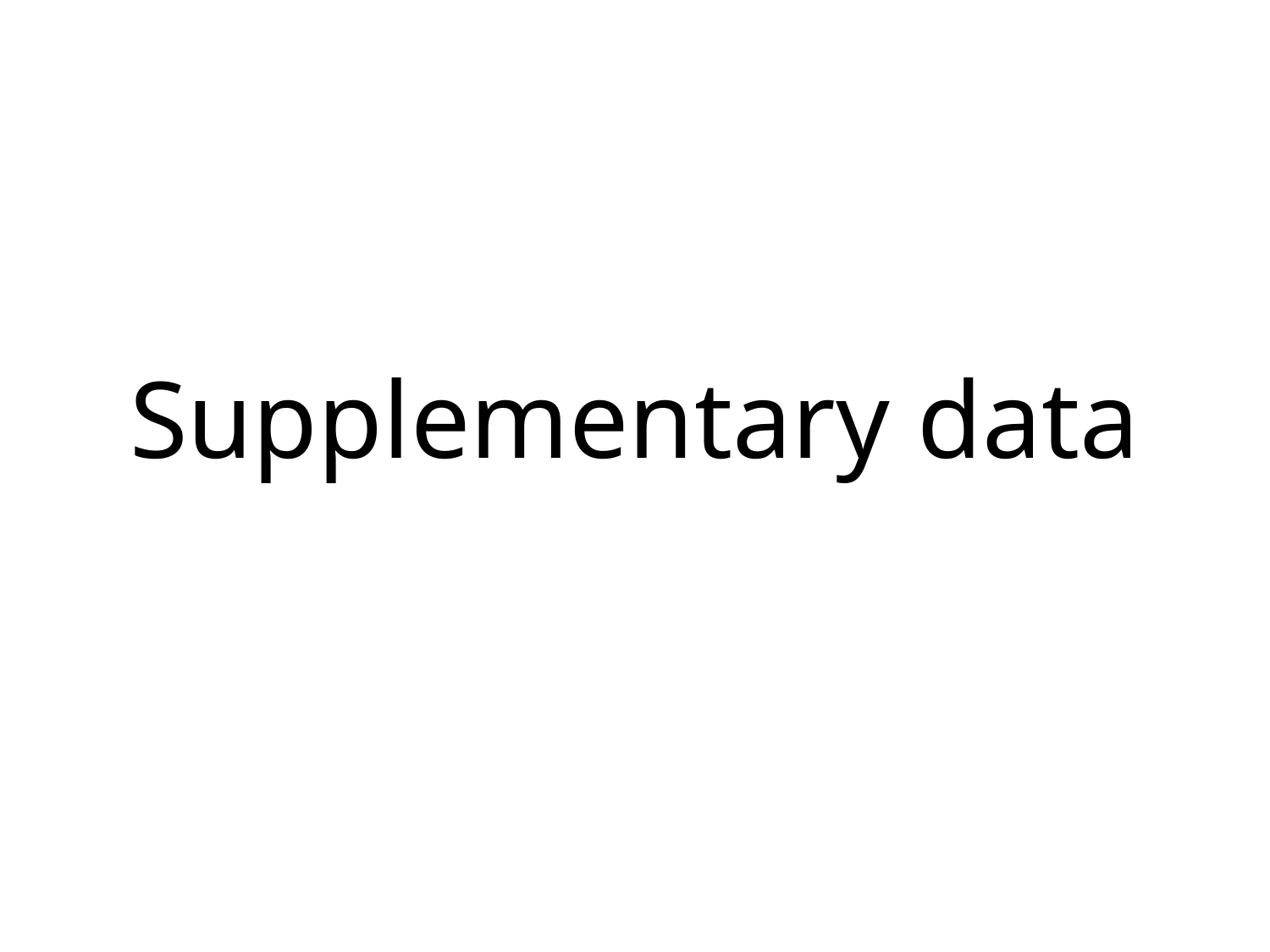

# Supplementary data

## Slide 2
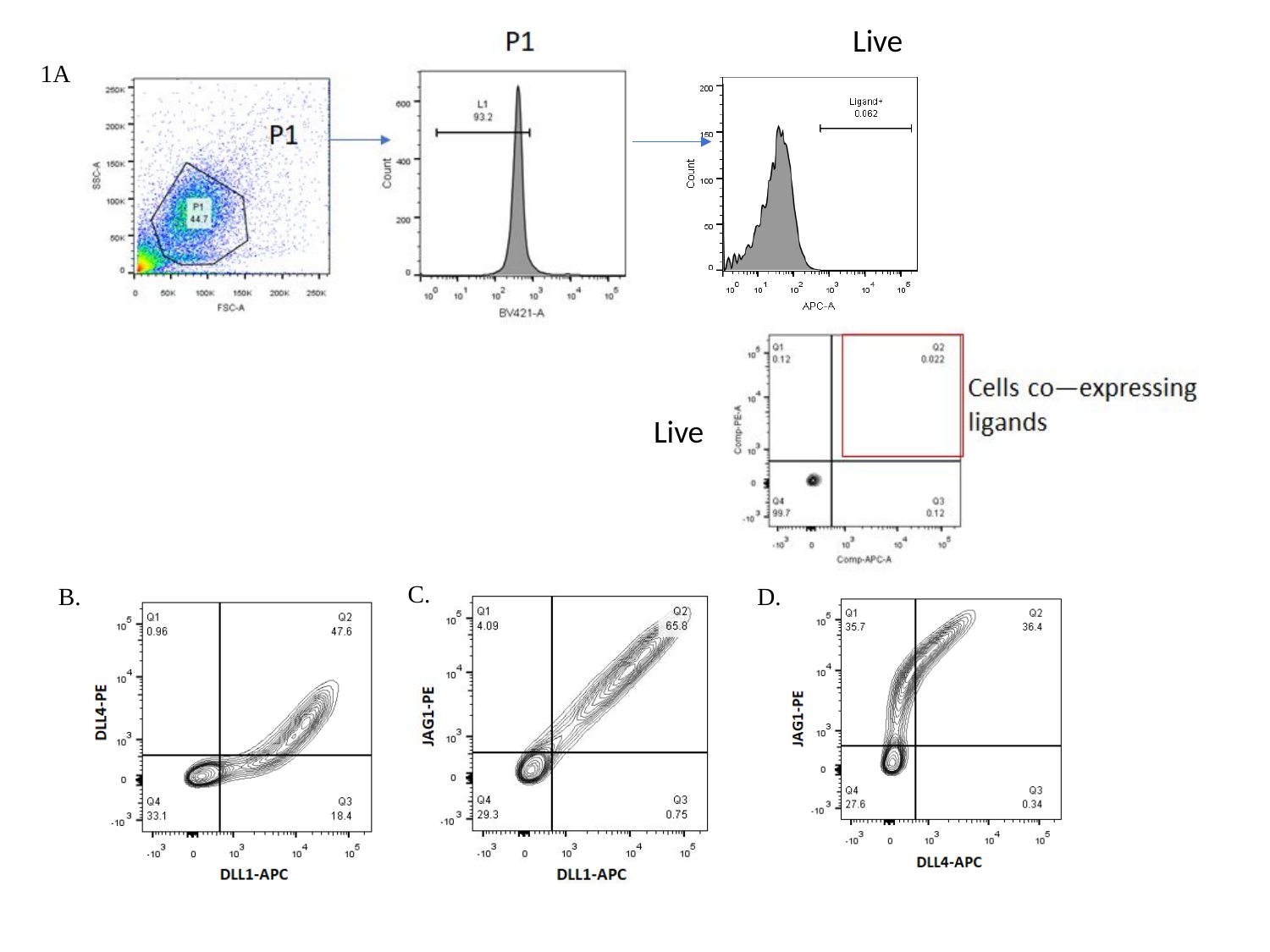

Live
1A
Live
C.
D.
B.

## Slide 3
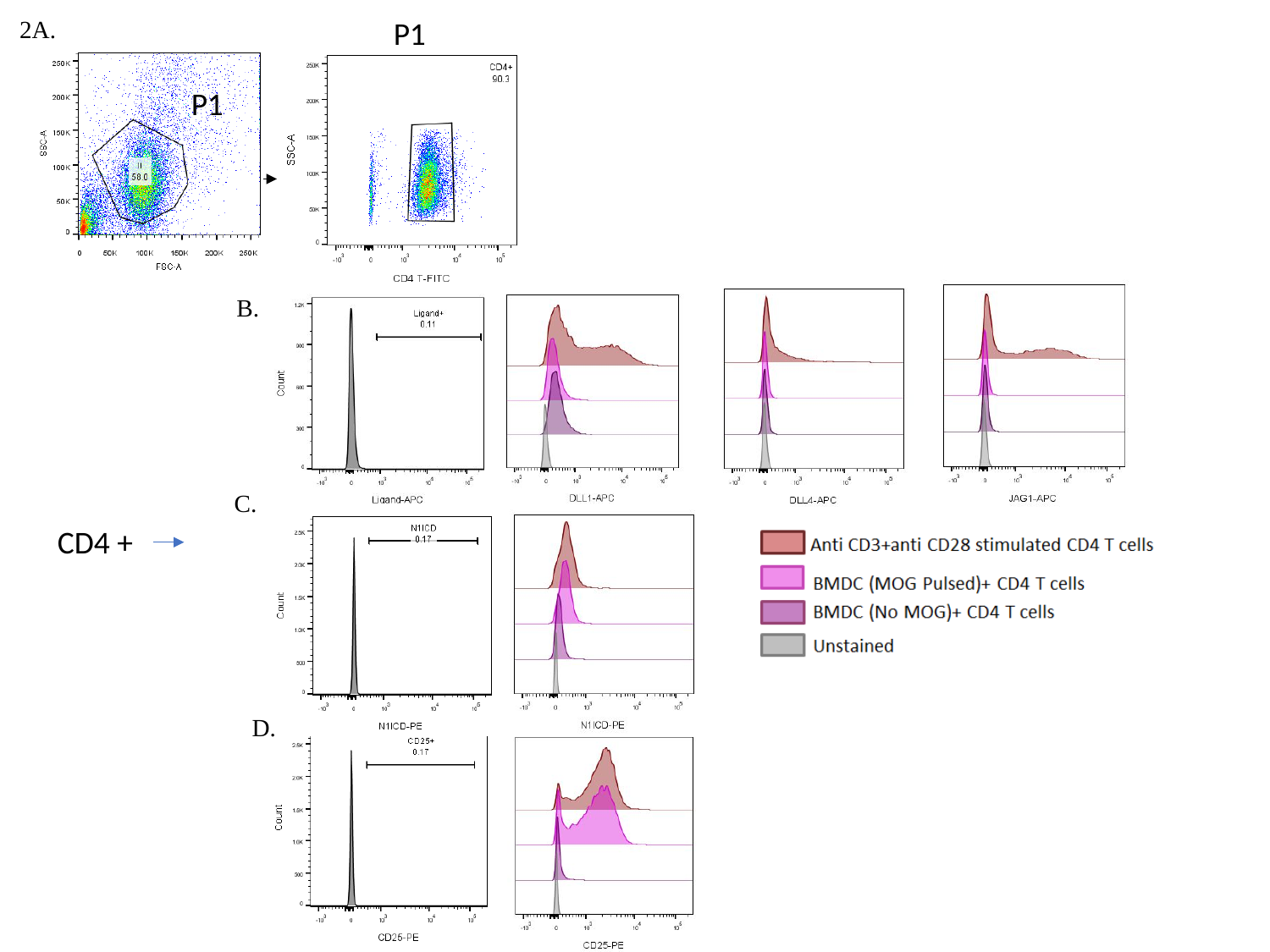

2A.
P1
P1
 B.
 C.
CD4 +
 D.

## Slide 4
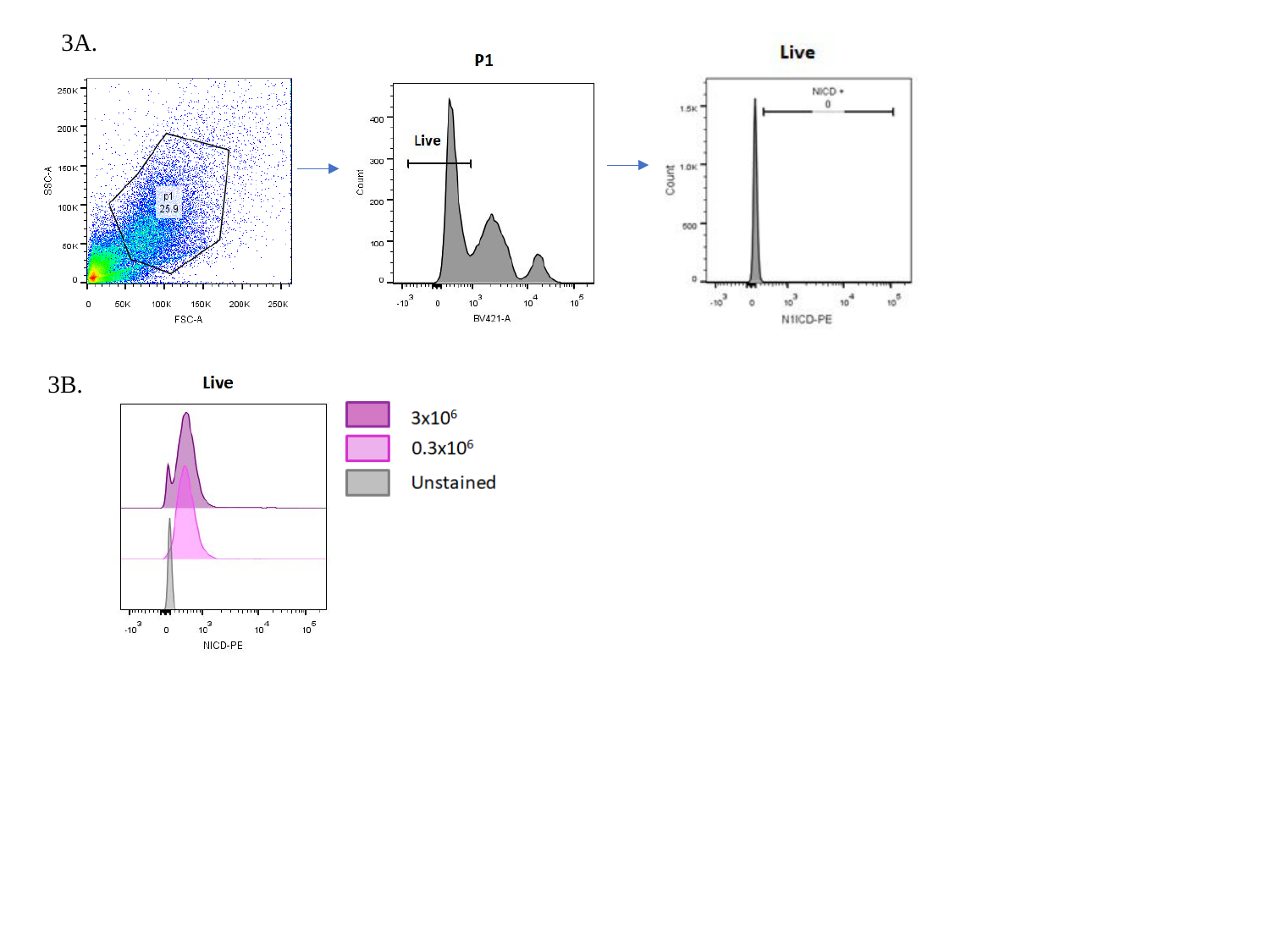

3A.
3B.

## Slide 5
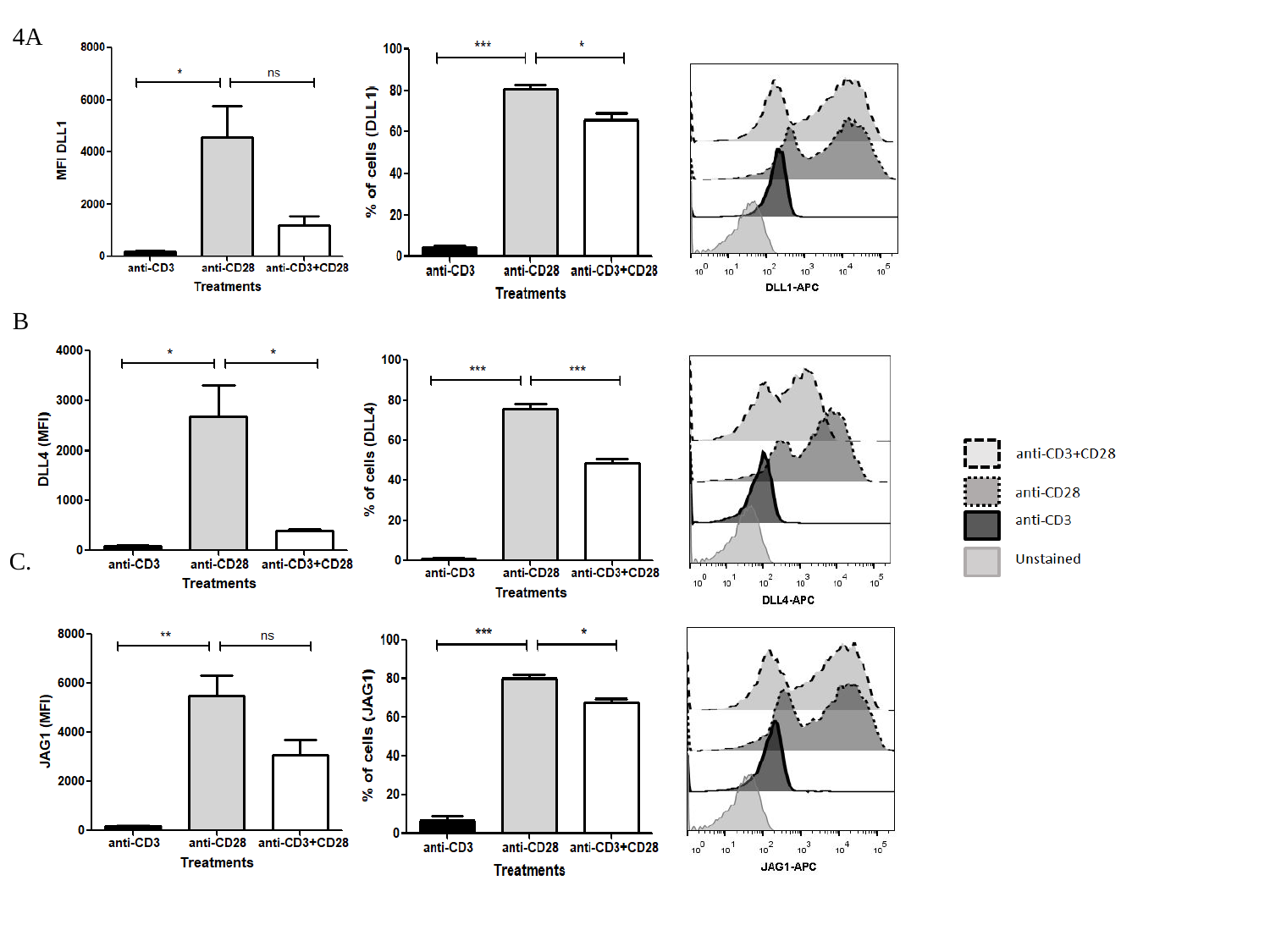

4A
B
C.

## Slide 6
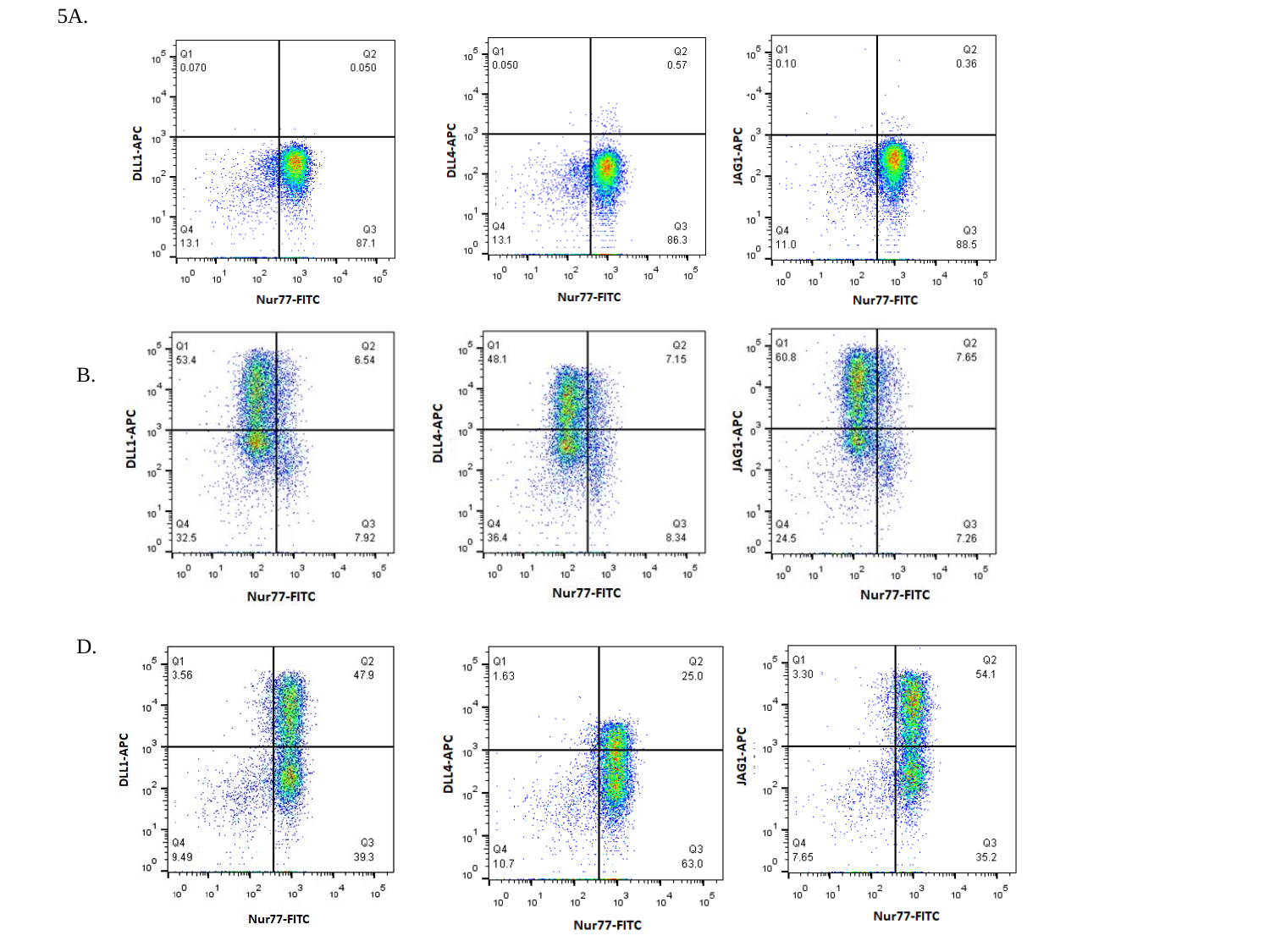

5A.
B.
D.

## Slide 7
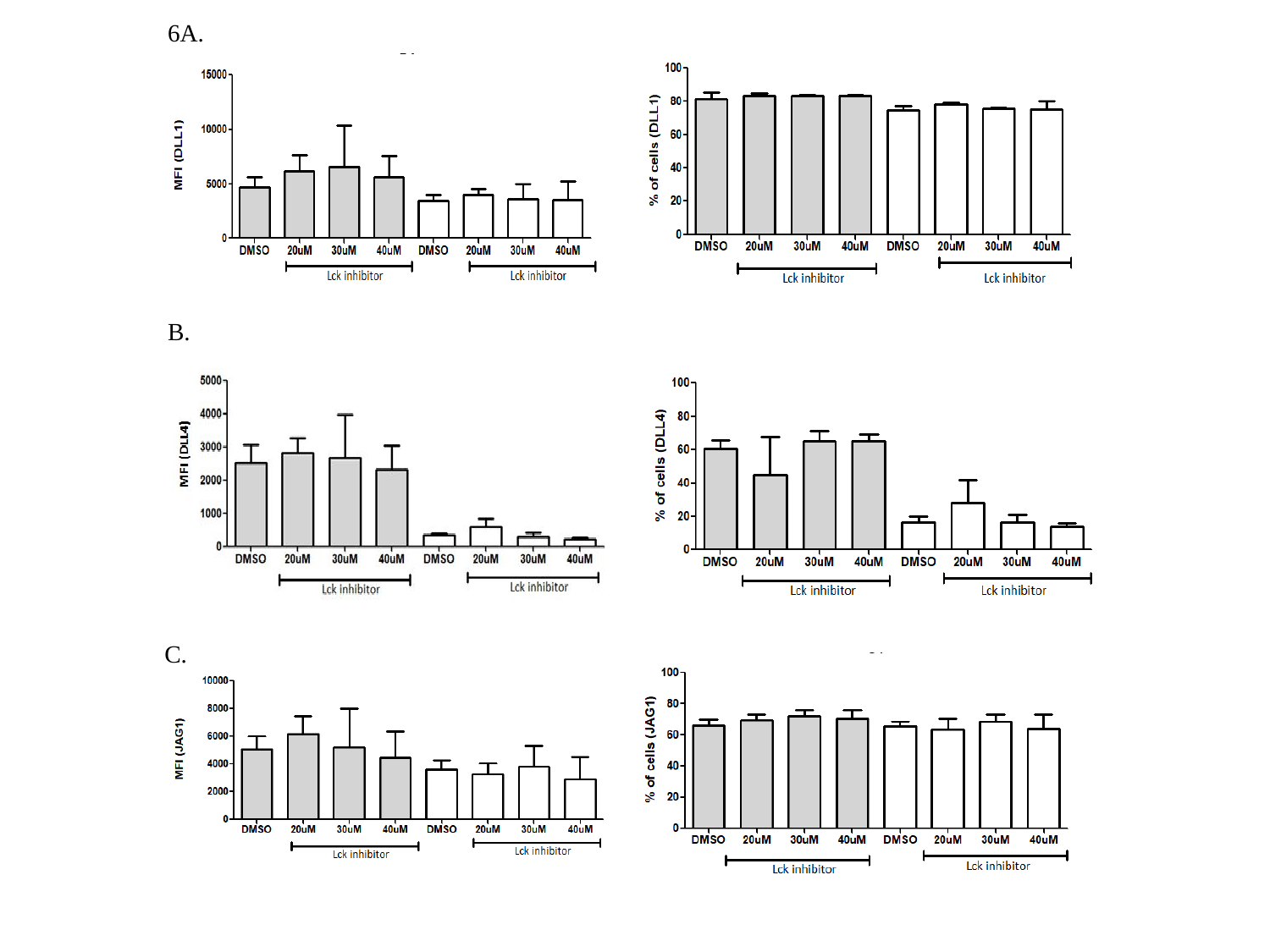

6A.
B.
C.

## Slide 8
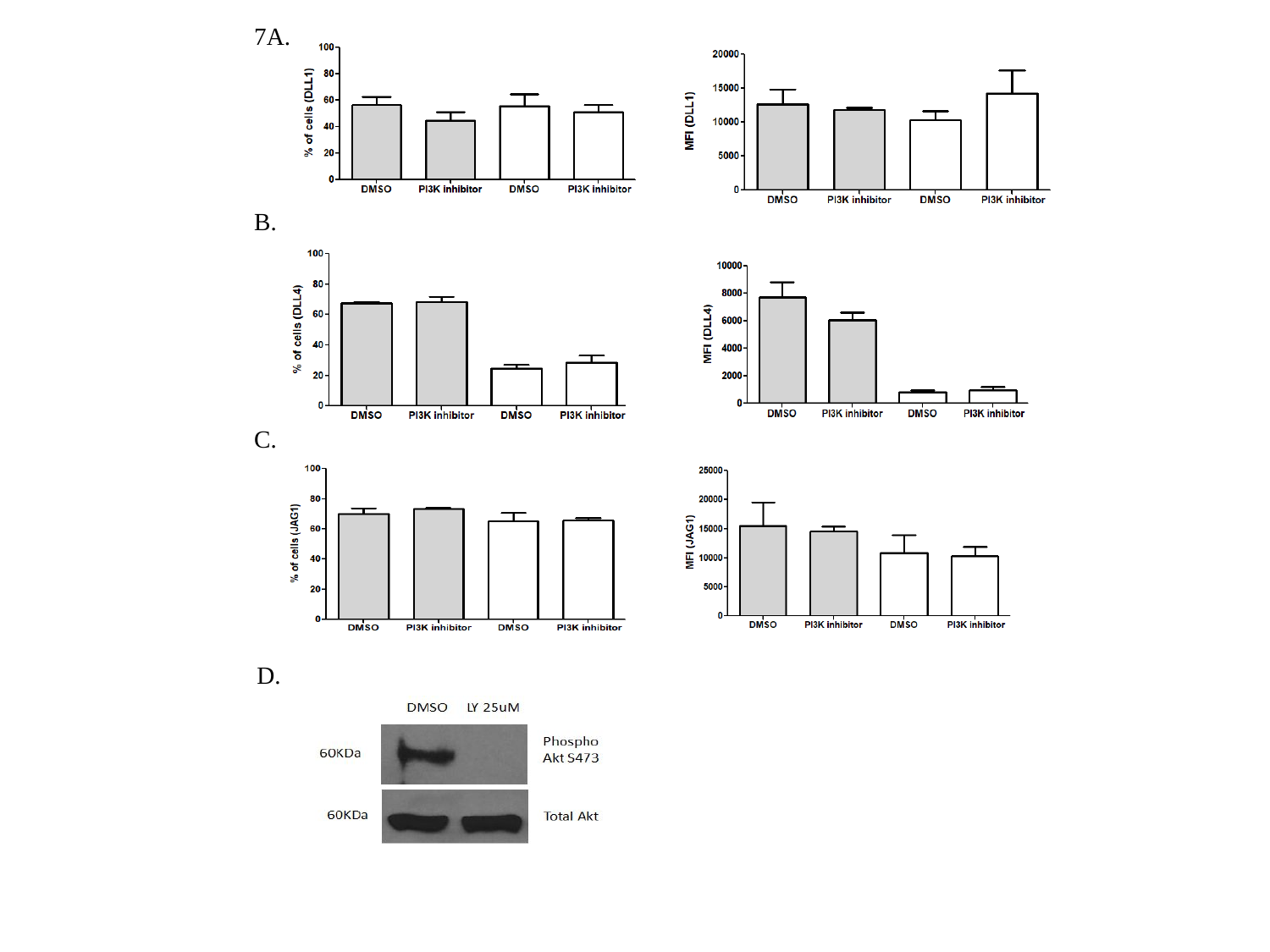

7A.
B.
C.
D.

## Slide 9
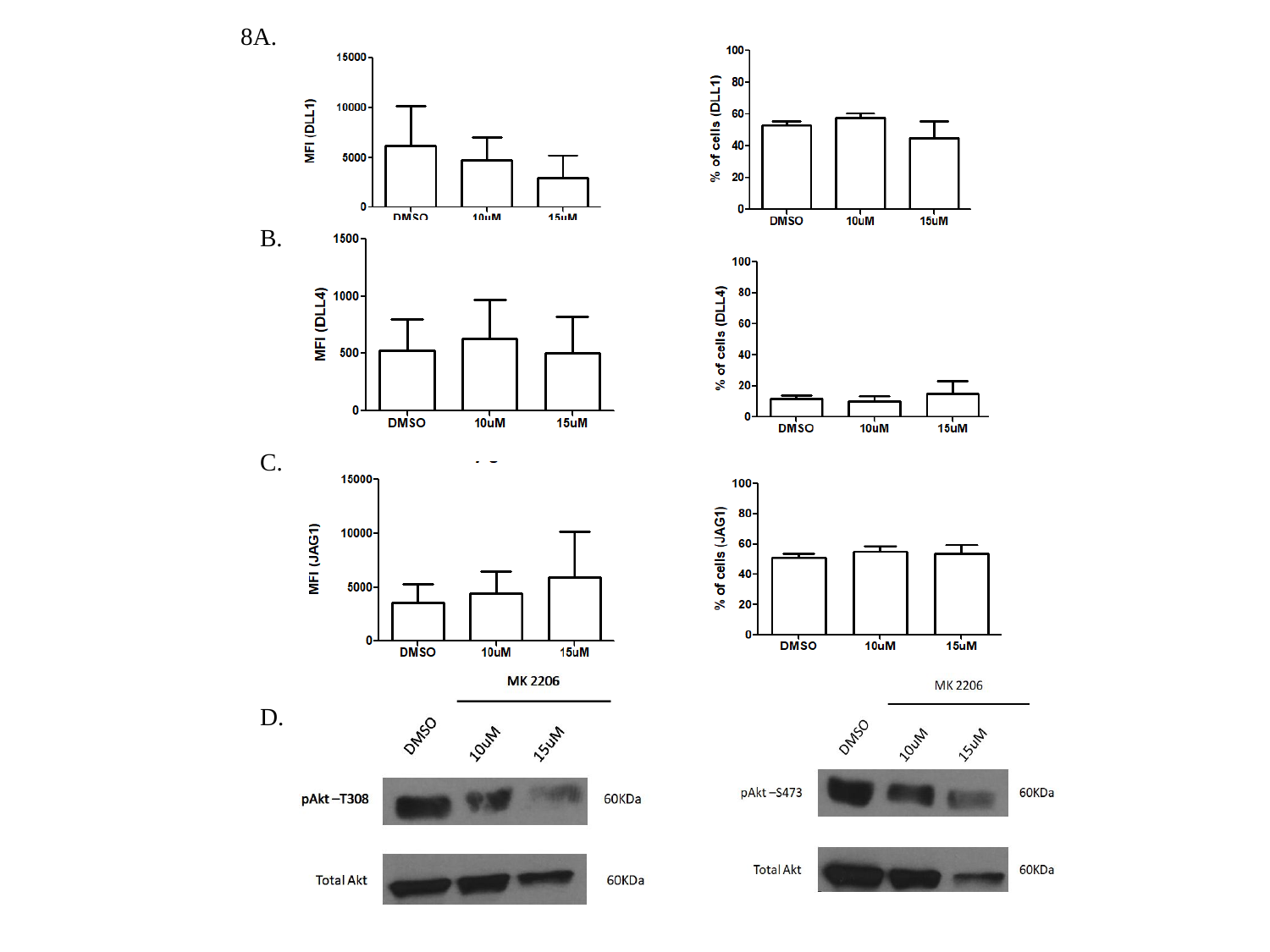

8A.
B.
C.
D.

## Slide 10
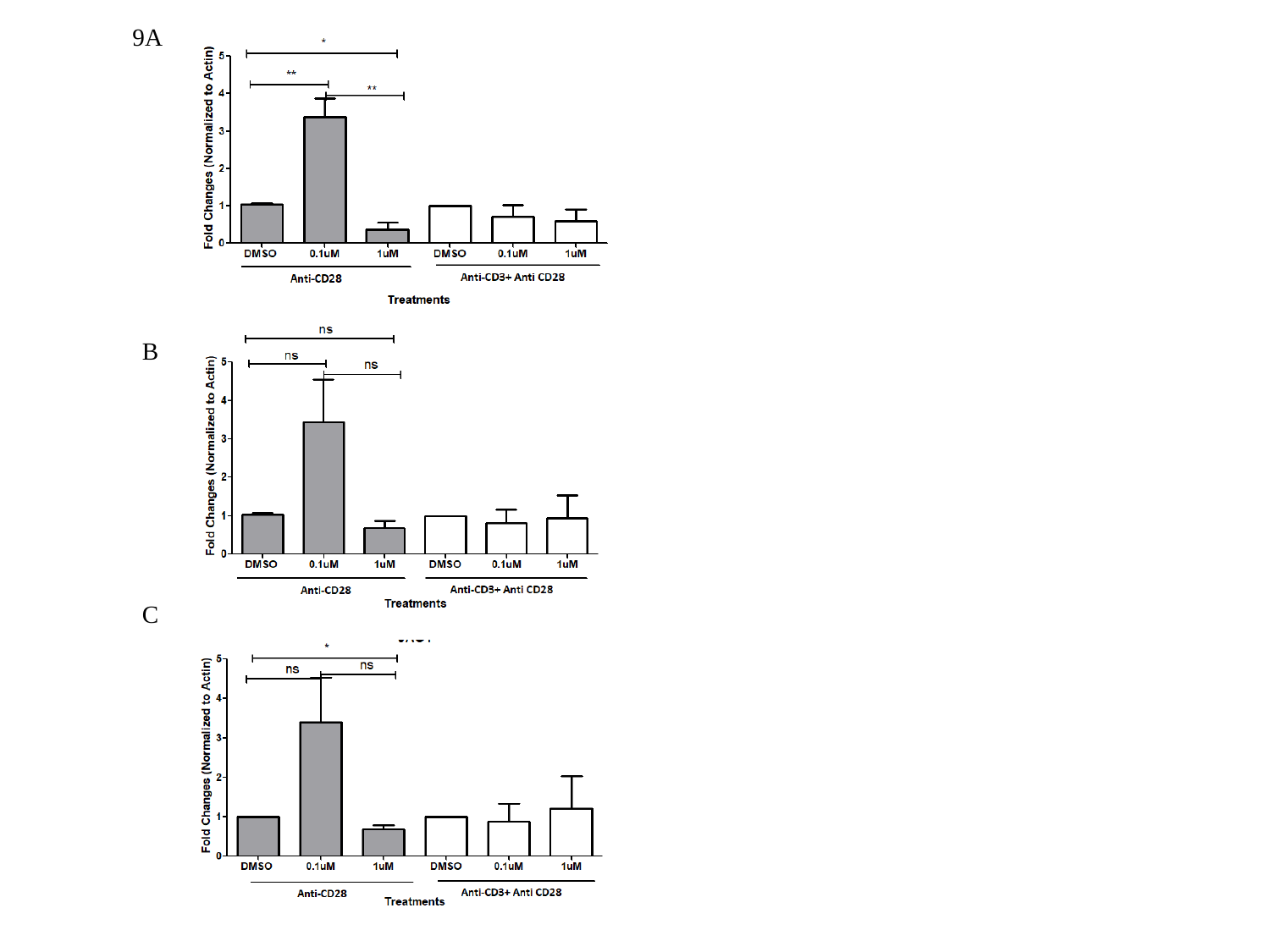

9A
B
C

## Slide 11
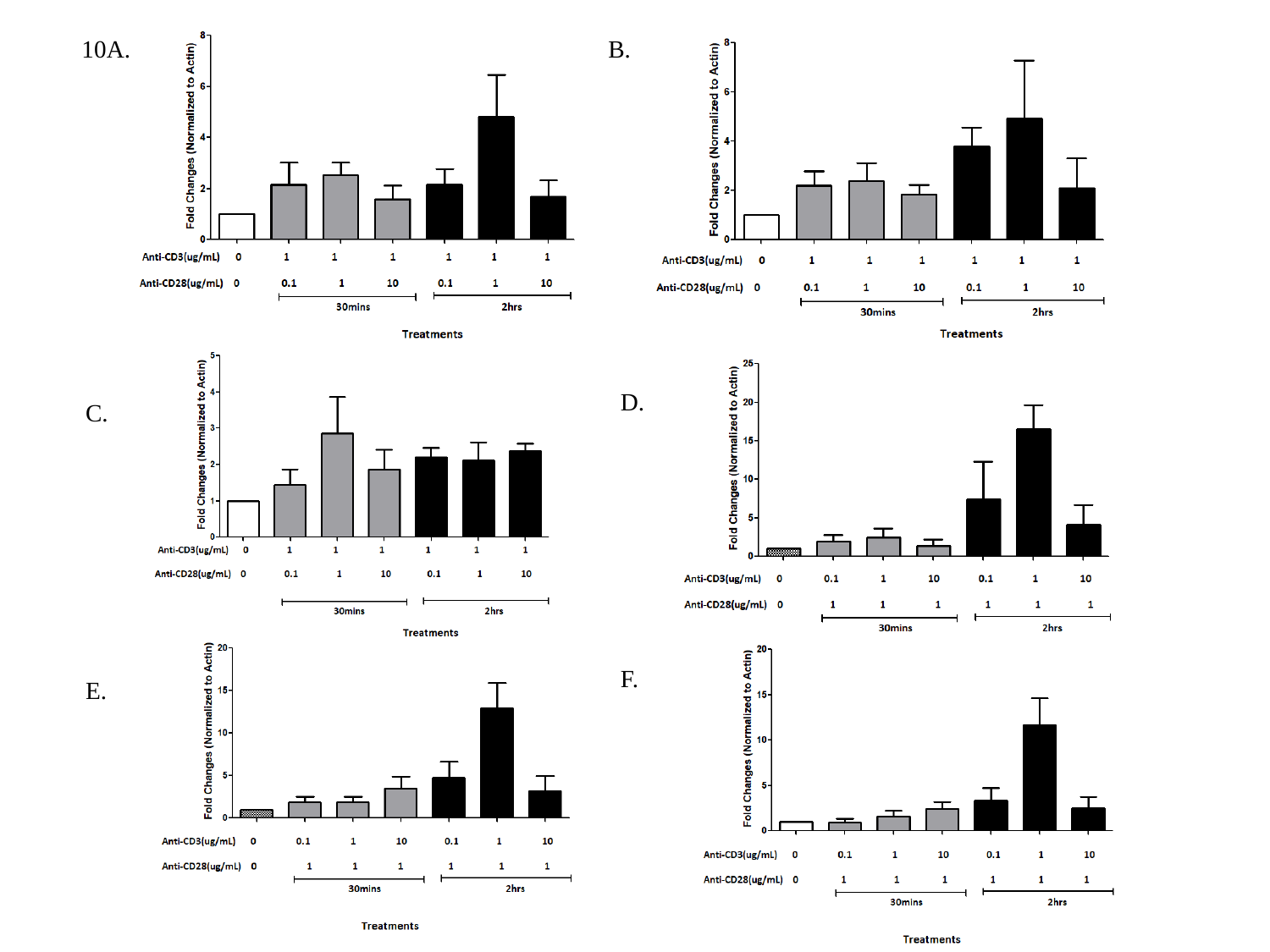

B.
10A.
D.
C.
F.
E.

## Slide 12
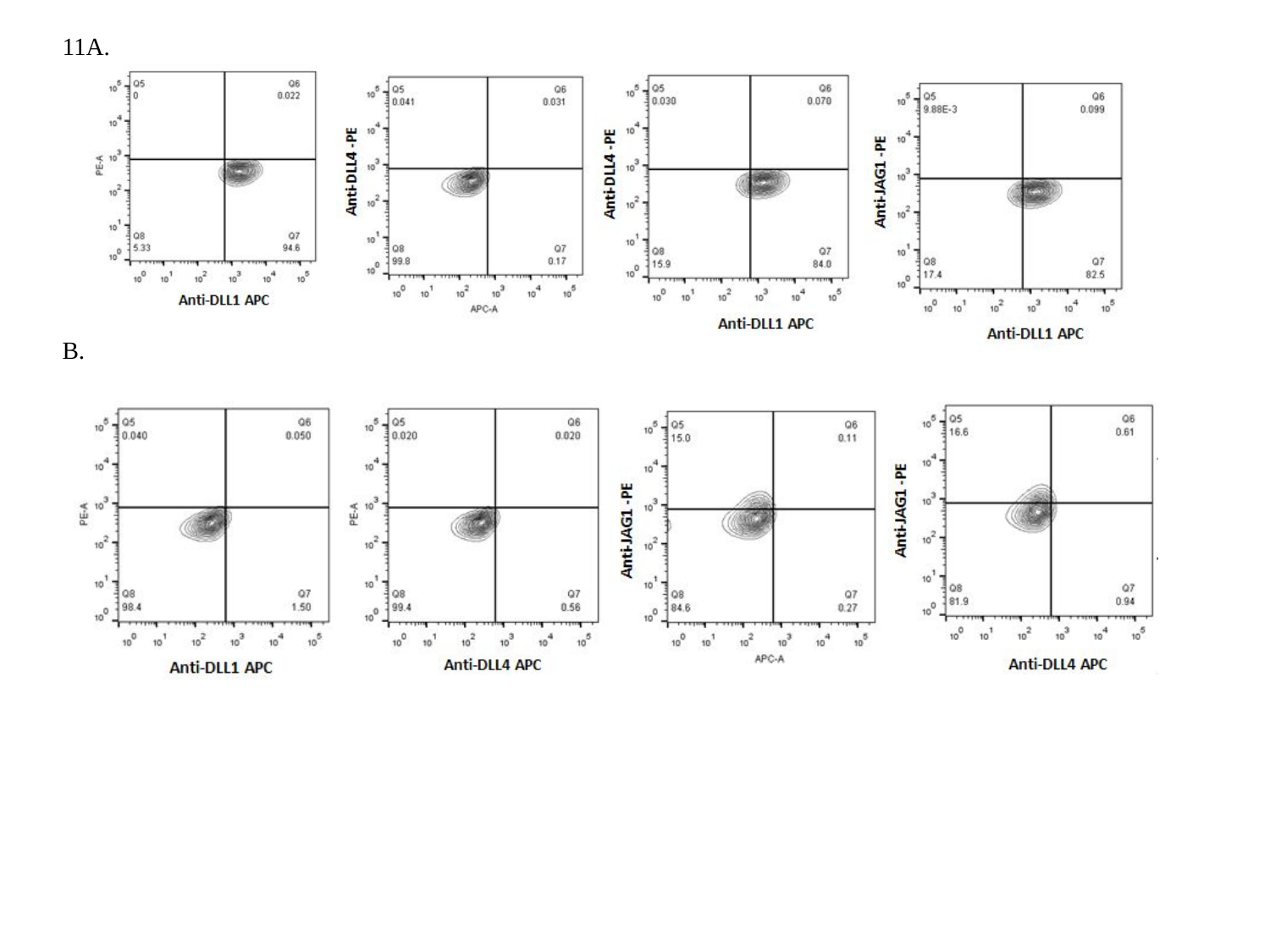

11A.
B.
