# Supplementary material for: CD28 Signaling Drives Notch Ligand Expression on CD4 T Cells
Source: Front Immunol. 2020 May 7;11:735. doi: 10.3389/fimmu.2020.00735 (PMC7221189; doi:10.3389/fimmu.2020.00735)
Supplement: Supplementary file 2 [file Data_Sheet_1.docx]

**Supplementary material**

**Methods**

**Western Blot**

Whole cell lysates were prepared in RIPA buffer (150mM NaCl, 1% Ige Cal-CA360,0.1%SDS,50mMTris, pH-8.0,0.5% Sodium deoxycholate). Lysates were resolved on 8% SDS-PAGE gel. Protein was transferred on a nitro-cellulose membrane and blocked in Blotto (5% non-fat-dry milk, 0.2% Tween-20 in PBS). Membranes were probed over-night with primary antibody. Membranes were washed and incubated with horse-radish peroxidase (HRP) labeled secondary antibody. Membranes were developed using ECL reagents (Biorad). Primary antibodies used :anti-mouse Akt (pan) (C67E7), Phospho-Akt (Thr308) (244F9) and Phospho-Akt (Ser473) (Cell Signaling Technologies) , anti-Vinculin (Proteintech). Secondary antibodies: anti-Rabbit-HRP (Cell Signaling Technologies).

**Cell lines**

CHO cell lines stably expressing either full-length Delta-like-1 (CHO-DLL1) or full-length Jagged1 (CHO-Jagged1) were a generous gift from Dr. Raphael Kopan (Cincinnati Children’s Hospital). All APC cells were maintained in RPMI, supplemented with 10% FBS, L-Glutamate, sodium pyruvate, penicillin/streptomycin and b-mercaptoethanol.

**RNA isolation and quantitative RT-PCR**

RNA was isolated from cells with the Quick-RNA Mini-Prep kit (Zymo Research, Irvine, CA). cDNA was synthesized with Oligo(dt) 12-18 primer (ThermoFisher Scientific, Waltham, MA), m-MLV reverse transcriptase (Promega Corporation, Madison, WI), and dNTPs (New England Biolabs, Ipswich, MA). cDNA was used in qRT-PCR reactions with SYBR Green Master Mix (Bimake, Houston, TX). Reaction was conducted on a Stratagene Mx3000p (Agilent Technologies, Santa Clara, CA). Primer sequences (Integrated DNA Technologies, Coralville, IA) were used for qPCR are listed in Table1. Transcripts were quantified using the 2-ΔΔCT CT (Livak) method. The primers 5’-CAGGACCTTCTTTGCCGTATG-3’ and 5’-AAGGGGAATCGGATG GGGTT-3’ were used to amplify DLL1 cDNA. The primers 5’-TTCCAGGCAACCTTCTCC GA-3’ and 5’-ACTGCCGCTATTCTTGTCCC-3’ were used to amplify DLL4 cDNA. The primers 5’-CCTCGGGTCAGTTTGAGCTG-3’ and 5’-CCTTGAGGCACACTTTGAAGTA-3’were used to amplify Jagged1 cDNA. The primers 5’-GGCTGTATTCCCCTCCATCG-3’ and 5’-CCAGTTGGTAACAATGCCATGT-3’’were used to amplify Actin cDNA.

**Figure S1 Gating strategy for Notch ligand expression on CD4 T cells:** CD4 T cells from C57BL/6 mice were treated with soluble anti-CD3ε and anti-CD28 at 1ug/mL each for indicated time points. Cells were harvested and analyzed by flow cytometry (A) Primary CD4 T cells population was selected (P1), followed by gating on live cell population (Live) as determined by absence of Zombie staining. Live population not stained with antibodies against Notch ligands (PE or APC tagged) was used to determine gate for Ligand + cells. (B-D) Contour plot representation of the co-expression of Notch ligands DLL1 and DLL4, DLL1 and JAG1 and DLL4 and JAG1.

**Figure S2 Gating for BMDC+CD4 T cell co-culture** CD4 T cells from 2D2 Transgenic mice were cocultured with MOG_35-55_ pulsed mature bone marrow derived dendritic cells (BMDC) obtained from 2D2 mice. T cells were stained with CFSE and BMDCs with Cell trace violet cell tracker dye before setting up for coculture. CD4 T cells stimulated with anti CD3ε or anti CD28 or anti CD3ε and anti-CD28, at 1ug/ml each was activated along with the coculture. Cells harvested after 6hours and analyzed by flow cytometry for ligands and N1ICD and after 24hours for CD25. (A) Gating strategy of primary population and FITC+ CD4 T cell population. Histograms for (B) DLL1, DLL4, JAG1 (C) Notch1ICD (Intracellular Notch1) and (D) CD25 expression on CD4 T cells. Data is a representative of three independent experiments. Data is a representative of three independent experiments

**Figure S3 Gating strategy and overlay for N1ICD+ cells:** CD4 T cells from C57BL/6 mice were stimulated with soluble anti-CD3ε and anti-CD28 at 1ug/mL at a concentration of 0.3x10^6^ or 3x10^6^ for 24hours. Cells were harvested after 24 hours and intracellularly stained for Notch1 ICD. (A)Primary CD4 T cells population was selected (P1), followed by gating on live cell population (Live) as determined by absence of Zombie staining. Live population not stained for N1ICD was used to determine gate for N1ICD + cells. (B) Overlay for N1ICD stained cells. Data is a representative of three independent experiments.

**Figure S4 Signaling through CD3 suppresses Notch ligand expression:** CD4 T cells from Nur77 mice were treated with either soluble anti-CD3ε or anti-CD28 or anti-CD28 and anti-CD3ε together for 6hours. Cells were harvested and analyzed by flow cytometry by gating on live cell population as determined by absence of Zombie staining. MFI values and percentage of ligand positive cells were plotted for (A) DLL1, (B) DLL4, (C) JAG1. Histograms to the right of (A-C) show expression of DLL1, DLL4 and JAG1 with indicated treatments. Data represents three independent experiments MFI value. Data represent mean ± SEM. *p <0.05, **p <0.005 and ***p <0.001. ns, not significant

**Figure S5 Dot plots representative of flow cytometry:** CD4 T cells from Nur77 mice were treated with either soluble anti-CD3ε or anti-CD28 or anti-CD28 and anti-CD3ε together for 6hours. Cells were harvested and analyzed by flow cytometry by gating on live cell population as determined by absence of Zombie staining. Representative plots for (A) anti-CD3ε treatment only, (B) anti-CD28 treatment only and (C) anti-CD3ε and anti-CD28 treatment for CD4 T cells obtained from Nur77 mice. Data is a representative of three independent experiments. Numbers in each quadrant represent percentage of cells.

**Figure S6** **Expression of Notch ligand on CD4 T cells is not mediated by Lck phosphorylation:** CD4 T cells from C57BL/6 mice were treated with soluble anti-CD28 alone or anti-CD3ε and anti-CD28 for 6 hours in the presence of Lck inhibitor at 20uM, 30uM, or 40uM or DMSO in the (Ctrl). Cells were harvested and analyzed by flow cytometry by gating on live cell population as determined by the absence of Zombie staining. MFI values and percentage of ligand positive cells were plotted for (A) DLL1, (B) DLL4, and (C) JAG1. Data represent three independent experiments. Data represent mean ± SEM.

**Figure S7 PI3K signaling downstream of CD28 does not regulate Notch ligand expression:** CD4 T cells from C57BL/6 mice were treated with soluble anti-CD28 alone or anti-CD3ε and anti-CD28 for 6 hours in the presence of PI3K inhibitor (LY294002) at 25uM or DMSO in the (Ctrl). Cells were harvested and analyzed by flow cytometry by gating on live cell population as determined by the absence of Zombie staining. MFI values and percentage of ligand positive cells were plotted for (A) DLL1, (B) DLL4, and (C) JAG1. Western blot performed to test LY294002 action on phosphorylation of Akt (D) Blockade of Akt phosphorylation at S473 by LY294002. Data represents three independent experiments. Data represent mean ± SEM.

**Figure S8 Akt signaling downstream of CD28 does not regulate Notch ligand expression:** CD4 T cells from C57BL/6 mice were treated with soluble anti-CD3ε and anti-CD28 for 6 hours in the presence of Akt inhibitor (MK2206) at 10uM or 15uM or DMSO in the (Ctrl). Cells were harvested and analyzed by flow cytometry by gating on live cell population as determined by the absence of Zombie staining. MFI values and percentage of ligand positive cells were plotted for (A) DLL1, (B) DLL4, and (C) JAG1. (D) reduced phosphorylation of Akt by MK2206. Data represents three independent experiments. Data represent mean ± SEM.

**Figure S9** **Transcript levels of Notch ligands do not show dose dependent response to NFκB inhibition:** CD4 T cells from C57BL/6 mice were stimulated with soluble anti-CD28 only or anti-CD3ε and anti-CD28 for 2hours in the presence of NFκB inhibitor BAY11 at 0.1uM or 1uM or DMSO as control. Quantitative RT-PCR analysis of the Notch ligands (A) DLL1 (*Dll1*) (B) DLL4 (*Dll4*) and (C) JAG1 (*Jagged1*) in CD4 T cells upon BAY11 treatment with DMSO control, presented relative to results obtained for the *Actin* (control). Data represent three independent experiments. Data represents mean ± SEM. *p <0.05, **p <0.005 and ns, not significant

**Figure S10 Transcript levels of Notch ligands do not show dose dependent response to titrated amount of anti-CD3ε and anti-CD28:** CD4 T cells from C57BL/6 mice were stimulated with soluble anti-CD3ε and anti-CD28 in the indicated amounts for 30mins and 2hours. Quantitative RT-PCR analysis of the Notch ligands (A) DLL1 (*Dll1*) (B) DLL4 (*Dll4*) and (C) JAG1 (*Jagged1*) in CD4 T cells upon stimulation with indicated amounts of anti-CD28 with 1ug/mL of anti-CD3ε for 30mins and 2hours, presented relative to results obtained for the *Actin* (control). Quantitative RT-PCR analysis of the Notch ligands (D) DLL1 (*Dll1*) (E) DLL4 (*Dll4*) and (F) JAG1 (*Jagged1*) in CD4 T cells upon stimulation with indicated amounts of anti-CD28 with 1ug/mL of anti-CD3ε for 30mins and 2hours, presented relative to results obtained for the *Actin* (control). Data represents three independent experiments. Data represent mean ± SEM.

**Figure S11 Testing antibodies for DLL1, DLL4 and JAG1**: (A) CHO-DLL1 cells stained with anti-DLL1-APC alone, anti-DLL4-PE alone, combination of anti-DLL1-APC and anti-DLL4-PE or combination of anti-DLL1-APC and anti-JAG1-PE. (B) CHO-Jagged1 cells were stained with anti-DLL1-APC alone, anti-DLL4-APC alone, anti-JAG1-PE alone or a combination of anti-JAG1-PE and anti-DLL4-APC.
